# Supplementary material for: Neighbourhood topology unveils pathological hubs in the brain networks of epilepsy-surgery patients
Source: Brain Commun. 2025 Oct 31;7(6):fcaf431. doi: 10.1093/braincomms/fcaf431 (PMC12646151; doi:10.1093/braincomms/fcaf431)
Supplement: fcaf431_Supplementary_Data [file fcaf431_supplementary_data.pdf]

# Supplementary information to: Neighbourhood topology unveils pathological hubs in the brain networks of epilepsy-surgery patients

Leonardo Di Gaetano,[1] Fernando A.N. Santos,[2] Federico Battiston,[1] Ginestra Bianconi,[3,4] Nicolò Defenu,[5] Ida Nissen,[6] Elisabeth C. W. van Straaten,[6,7] Arjan Hillebrand,[6,8,9] and Ana P. Millán[6,10]

[1]*Department of Network and Data Science, Central European University, 1100 Vienna, Austria*

[2]*Institute for Advanced Study, University of Amsterdam, Amsterdam, 1012 GC, The Netherlands*

[3]*School of Mathematical Sciences, Queen Mary University of London, Mile End Road, E1 4NS, London, United Kingdom*

[4]*The Alan Turing Institute, British Library, 96 Euston Road, NW1 2DB, London, United Kingdom*

[5]*Institute for Theoretical Physics, ETH Zürich, Wolfgang-Pauli-Str. 27, 8093 Zurich, Switzerland*

[6]*Amsterdam UMC, Vrije Universiteit Amsterdam, Department of Clinical Neurophysiology and MEG Center, De Boelelaan 1117, Amsterdam, The Netherlands*

[7]*Academic Center for Epileptology Kempenhaeghe and MUMC+, Sterkselseweg 65, 5591 VE Heeze, the Netherlands*

[8]*Amsterdam Neuroscience, Brain Imaging, Amsterdam, The Netherlands*

[9]*Amsterdam Neuroscience, Systems & Network Neuroscience, Amsterdam, The Netherlands*

[10]*Department of Electromagnetism and Matter Physics, and Institute "Carlos I" of Statistical and Computational Physics, University of Granada, Granada, Spain**

## S.1. PATIENT CHARACTERISTICS

The patient cohort included both patients with temporal ($n = 61$) and extra-temporal ($n = 30$) resections. Respectively 77.05% of patients with a temporal resection ($n = 47$) and 50.00% of patients with an extra-temporal resection ($n = 15$) had a good outcome. Just under half of the patients were female ($n = 43$). Respectively 72.09% of female patients and 64.58% of male patients had a good outcome. The data is detailed in Supplementary Table 1.

| | Total | Female | Temporal Resection | Female & Temporal Resection |
|---|---|---|---|---|
| SF | 62 | 31 | 47 | 26 |
| NSF | 29 | 12 | 14 | 8 |
| Cohort | 91 | 43 | 61 | 34 |

Supplementary Table 1: Summary of patient characteristics, separately for patients with good (SF) and bad (NSF) outcome, as well as for the full cohort. We indicate the number of female patients, the number of patients with temporal resections, and the number of female patients with temporal resections.

## S.2. DISPARITY FILTER: BASIC NETWORK STATISTICS

The brain networks were thresholded with a disparity filter with significance threshold of $\alpha = 0.1$. In Supplementary Table 2 we report the average number of edges remaining in the network after the thresholding procedure, and the size of the giant component.

## S.3. BASIC NODE-SETS STATISTICS

In Supplementary Figure 1 we report the distribution of node-set sizes for each frequency band.

* apmillan@ugr.es

| Band | $B$ | $\delta$ | $\theta$ | $\alpha_1$ | $\alpha_2$ | $\beta$ | $\gamma$ |
|---|---|---|---|---|---|---|---|
| $L$ | 384.15 | 416.63 | 393.74 | 395.90 | 403.04 | 385.15 | 392.84 |
| $S$ | 84.49 | 89.91 | 89.09 | 89.32 | 89.38 | 89.10 | 89.81 |

Supplementary Table 2: Basic network statistics. Average number of edges $L$ remaining in the network after the thresholding procedure, and the average size of the largest component $S$, for each frequency band.

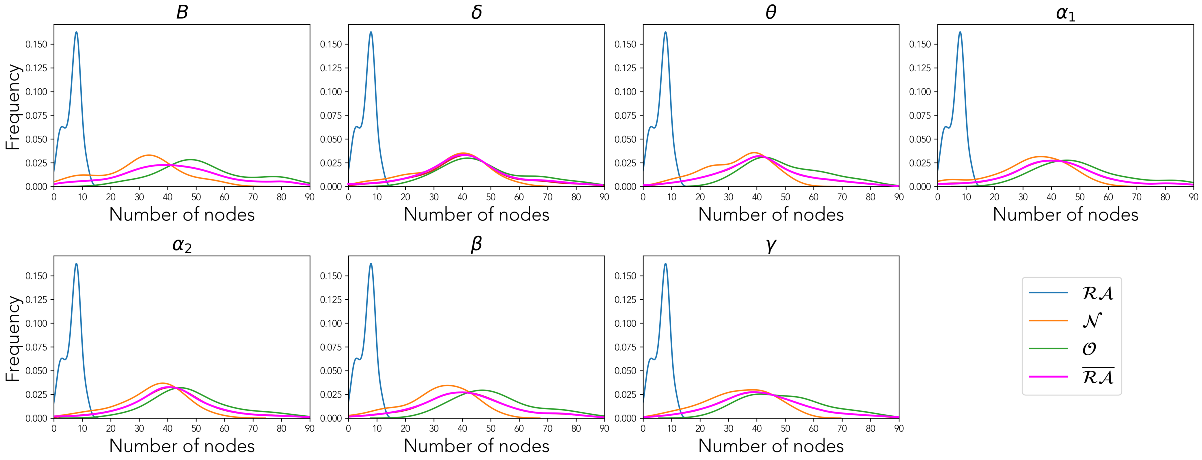


Supplementary Figure 1: Distributions of the size of each node-set (as indicated by the general legend) over the patient population, for each frequency band as indicated by the panel title.

## S.4. METRICS OF NETWORK TOPOLOGY

To quantify the connectivity and network properties of brain nodes, we utilized a variety of metrics. These metrics can be categorized into node-level metrics and neighbourhood-level metrics. Below is a detailed description of each metric:

### A. Node-level Metrics

- The **Betweenness Centrality** $BC$ measures the influence of a node over the flow of information within the network. It is calculated by determining the fraction of all shortest paths in the network that pass through a given node. Nodes with high betweenness centrality are considered critical for information transfer and can be identified as hubs within the network [1].

- The **Local Clustering Coefficient** $c$ quantifies the extent to which nodes in a graph tend to form clusters or groups. For a given node $i$, the clustering coefficient is defined as the ratio of the number of closed triplets (or triangles) to the total number of triplets (both open and closed) centered on that node. Mathematically, it is given by:

$$c_i = \frac{2 \times \text{Number of closed triangles including node } i}{k_i(k_i - 1)} \tag{1}$$

  where $k_i$ is the degree of node $i$. A higher clustering coefficient indicates a greater tendency for node $i$ to form tightly-knit groups with its neighbors [1].

- The **Local Curvature** $\mathcal{C}$ captures how paths bend around a node in its vicinity, offering insights into the local geometric structure More specifically:

$$C_i = \sum_{k=1}^{k_{max}} (-1)^{k+1} \frac{Cl_{ik}}{k}, \tag{2}$$

  where $Cl_{ik}$ is the number of $k$-cliques to which $i$ belongs, and $k_{max}$ represents the size (i.e. number of nodes) of the largest clique in the network ($k_{max} = 3$ considering interactions up to three-node ones.). It generalizes the concept of curvature from differential geometry to network theory. Nodes with high curvature tend to have a significant influence on the robustness and stability of the network [2].

### B. Neighbourhood-level Metrics

The extended neighbourhood $\mathcal{EN}$ of a node encompasses all nodes within a certain distance (or hops) from the given node, excluding the node itself [3].

- The **Number of Nodes in the Extended Neighbourhood** $N$ measures the size of the $\mathcal{EN}$ and it generalizes the concept of node degree.
- The **Number of Edges in the Extended Neighbourhood** $E$ quantifies the total number of pairwise edges within the $\mathcal{EN}$, reflecting the local connectivity density.
- The **Betti Numbers ($\beta_0$, $\beta_1$, $\beta_2$)** are topological invariants that describe the connectivity of simplicial complexes (constructed in this case from the node neighbourhoods) at different dimensions, generalizing the notion of clustering coefficient:
  - $\beta_0$ represents the number of connected components in the $\mathcal{EN}$, indicating the degree of fragmentation. A higher $\beta_0$ indicates a node that acts as a broker between different communities.
  - $\beta_1$ quantifies the number of one-dimensional holes or open loops representing independent cycles within the $\mathcal{EN}$. It provides information on the presence of circular structures that are not filled in by higher-dimensional simplices.
  - $\beta_2$ measures the number of two-dimensional voids, reflecting higher-order connectivity patterns such as cavities within the $\mathcal{EN}$.

In the main section of this paper we used these 8 metrics to describe local and regional network organization for each node, for each patient- and frequency-specific brain network. In Supplementary Figure 2 we show an illustrative example of the distribution of values for each of these metrics, for each of the node-sets defined in the main text, namely nodes in and outside the resection area ($\mathcal{RA}$ and $\overline{\mathcal{RA}}$ node sets), the neighbours of the resection area $\mathcal{N}$ and other nodes in the network $\mathcal{O}$.

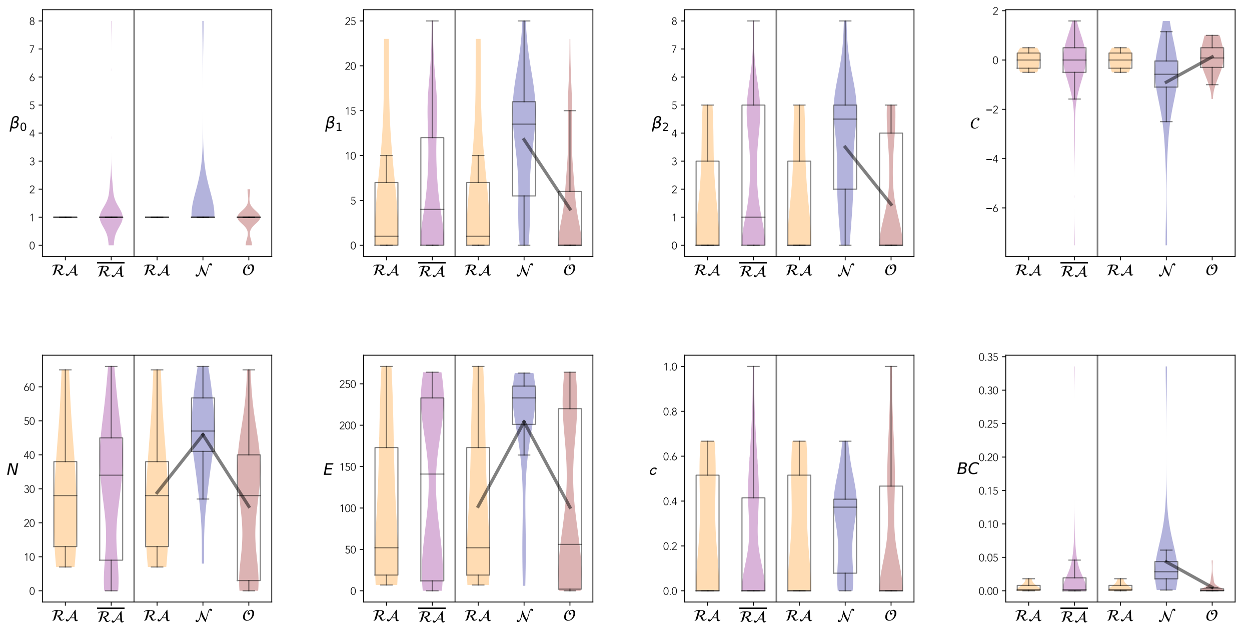


Supplementary Figure 2: Distribution of generalized centrality metrics for an exemplary case (SF patient, broadband network) for each node-set. Each panel corresponds to a generalized centrality metric as indicated by the labels. For each panel we show the results for the two analysis that were performed: the two-node-set partition (left) accounting for the $\mathcal{RA}$ and $\overline{\mathcal{RA}}$ node sets, and the three-node-set partition (right) accounting for the $\mathcal{RA}$, $\mathcal{N}$ and $\mathcal{O}$ node sets. In all panels we show the distribution of values for each node-set as a violin plot, and indicate the mean and median values with solid lines. The box-plots indicate the median, the 25% and 75% percentiles and the extreme values. Significant differences between two groups are indicated by black lines connecting the corresponding violins. The number of nodes in each group was, for all panels: $n(\mathcal{RA}) = 7$, $n(\overline{\mathcal{RA}}) = 83$, $n(\mathcal{N}) = 22$, $n(\mathcal{O}) = 61$. The results of the statistical testing (Wilcoxon rank-sum) are, from the top-left of the bottom-right panel: a) $(p, z) = (0.9 - 0.1)$, $(0.9, -0.2)$, $(0.8, 0.3)$, $(0.8, 0.3)$, $(0.9, -0.2)$, $(0.6, -0.5)$, $(0.9, 0.1)$, $(0.5, -0.7)$ for the $\mathcal{RA}$ vs $\overline{\mathcal{RA}}$ comparison; b) $(p, z) = (0.4, -0.8)$, $(0.10, -1.6)$, $(0.08, -1.8)$, $(0.2, 1.3)$, $(0.05, -2.0)$, $(0.02, -2.3)$, $(0.8, -0.3)$, $(0.14, -1.5)$ for the $\mathcal{RA}$ vs $\mathcal{N}$ comparison; c) $(p, z) = (0.5, 0.6)$, $(0.7, 0.4)$, $(0.9, 0.1)$, $(0.6, -0.5)$, $(0.7, 0.4)$, $(0.99, 0.01)$, $(0.8, 0.2)$, $(0.99, 0.02)$ for the $\mathcal{RA}$ vs $\mathcal{O}$ comparison; and d) $(p, z) = (0.09, 1.7)$, $(3.3 \cdot 10^{-5}, 4.2)$, $(9.9 \cdot 10^{-4}, 3.3)$, $(0.01, -2.5)$, $(2.0 \cdot 10^{-5}, 4.3)$, $(7.8 \cdot 10^{-5}, 4.0)$, $(0.3, 1.0)$, $(8.9 \cdot 10^{-3}, 2.6)$ for the $\mathcal{N}$ vs $\mathcal{O}$ comparison. Significant differences ($p < 0.05$) are shown in the figure via solid lines between the corresponding node groups.

## S.5. SUPPLEMENTARY INFORMATION TO THE PATIENT-SPECIFIC COMPARISONS

In Supplementary Tables 3 and 4 we report the data supporting Figure 3 in the main text. In particular, in Supplementary Table 3 we report the number of patients for whom there was a significant difference in the direction of the hypothesis in the node-comparison tests, for each of the four tests, and for each frequency band and generalized centrality metric. Similarly, in Supplementary Table 4 we report the same information regarding significant differences in the direction opposite to the hypothesis. In each case we also report within parentheses the number of these patients that belonged to the SF subgroup. The number of patients belonging to the NSF subgroup can be directly calculated by the difference.

In Supplementary Figure 3 we repeat the analysis in Figure 3 of the main text but specifically for the SF (top row) and NSF (bottom row) subgroups (see figure caption for details). We found similar results for the SF and NSF subgroups, with similar patterns of differences both in the direction of the hypotheses and against them. To identify if there were quantitative differences in the results, we compared the fraction of patients for whom there was a significant difference in the direction of the hypothesis or against it for each type of node test (see Supplementary Table 5), considering all generalized centrality metrics and frequency bands, and separately for the SF and NSF subgroups. Given that the curvature changes in opposite direction to the other metrics (i.e. more negative values are associated with hubness) as it can be seen in Supplementary Figure 3, the directions were inverted for this metric (as in the analyses in the main text). We measured whether there were significant differences between the SF and NSF subgroups at this level with the Wilcoxon signed-rank test for paired variables, and FDR (false discovery rate) corrected for multiple comparisons. We found that the comparison between $\mathcal{RA}$ and $\mathcal{N}$ differed significantly, with the NSF subgroup displaying significantly more differences, both in the direction of the hypothesis ($\mathcal{X}\,(\mathcal{RA} > \mathcal{N})$) and against it, than the SF group ($p = 0.003$ and $p = 0.034$ after FDR correction). The NSF subgroup also showed more negative differences (against the direction of the hypothesis) for the $\mathcal{X}\,(\mathcal{RA} > \mathcal{O})$ comparison than the SF group, but the difference did not survive FDR correction ($p = 0.075$ after the correction). Finally, the SF subgroup showed more positive differences for the $\mathcal{X}\,(\mathcal{N} > \mathcal{O})$ comparison than the NSF group ($p = 0.003$ after FDR correction). These results suggest that, for SF patients, $\mathcal{RA}$

nodes are more similar to their neighbours ($\mathcal{N}$ nodes), and that the latter are more central than the remaining nodes ($\mathcal{O}$ group), than for NSF patients.

Finally, we point out that the $p$-values in these analyses have been corrected for multiple comparisons within each patient analysis (Bonferroni correction). Given that $n = 91$ patients, we expected an average of 4.55 false positives on each test. We found 374 significant differences for $\mathcal{X}(\mathcal{RA}) > \mathcal{X}(\overline{\mathcal{RA}})$, 810 for $\mathcal{X}(\mathcal{RA}) > \mathcal{X}(\mathcal{O})$, 378 for $\mathcal{X}(\mathcal{RA}) > \mathcal{X}(\mathcal{N})$, and 1966 for $\mathcal{X}(\mathcal{N}) > \mathcal{X}(\mathcal{O})$.

(a) $\mathcal{X}(\mathcal{RA}) > \mathcal{X}(\overline{\mathcal{RA}})$

| | $BC$ | $c$ | $\mathcal{C}$ | $N$ | $E$ | $\beta_0$ | $\beta_1$ | $\beta_2$ |
|---|---|---|---|---|---|---|---|---|
| $B$ | 6(3) | 6(6) | 1(1) | 23(18) | 27(22) | 0(0) | 20(15) | 14(11) |
| $\delta$ | 3(0) | 0(0) | 1(1) | 4(1) | 6(2) | 1(0) | 6(2) | 2(2) |
| $\theta$ | 3(4) | 4(2) | 1(0) | 11(9) | 14(10) | 0(0) | 8(5) | 9(6) |
| $\alpha_1$ | 0(0) | 4(3) | 4(3) | 6(5) | 6(5) | 0(0) | 5(4) | 1(1) |
| $\alpha_2$ | 0(0) | 1(1) | 1(0) | 2(2) | 5(6) | 0(0) | 3(3) | 4(3) |
| $\beta$ | 0(0) | 5(2) | 4(1) | 4(3) | 5(3) | 0(0) | 4(3) | 3(2) |
| $\gamma$ | 1(0) | 0(0) | 2(2) | 5(2) | 4(1) | 0(0) | 4(2) | 1(1) |

(b) $\mathcal{X}(\mathcal{RA}) > \mathcal{X}(\mathcal{O})$

| | $BC$ | $c$ | $\mathcal{C}$ | $N$ | $E$ | $\beta_0$ | $\beta_1$ | $\beta_2$ |
|---|---|---|---|---|---|---|---|---|
| $B$ | 16(13) | 17(15) | 1(1) | 49(38) | 50(39) | 2(2) | 46(34) | 37(27) |
| $\delta$ | 5(1) | 4(3) | 0(0) | 15(8) | 18(11) | 1(0) | 15(8) | 5(3) |
| $\theta$ | 10(9) | 8(6) | 1(0) | 23(16) | 25(17) | 0(0) | 24(18) | 15(12) |
| $\alpha_1$ | 6(4) | 6(5) | 3(2) | 22(16) | 26(20) | 0(0) | 25(20) | 10(6) |
| $\alpha_2$ | 5(5) | 3(3) | 0(0) | 14(10) | 16(12) | 0(0) | 17(12) | 7(6) |
| $\beta$ | 4(4) | 9(6) | 4(0) | 17(10) | 21(14) | 0(0) | 17(11) | 9(5) |
| $\gamma$ | 5(2) | 1(1) | 1(1) | 11(6) | 12(6) | 0(0) | 9(4) | 2(1) |

(c) $\mathcal{X}(\mathcal{RA}) > \mathcal{X}(\mathcal{N})$

| | $BC$ | $c$ | $\mathcal{C}$ | $N$ | $E$ | $\beta_0$ | $\beta_1$ | $\beta_2$ |
|---|---|---|---|---|---|---|---|---|
| $B$ | 5(2) | 3(3) | 7(5) | 9(5) | 5(2) | 0(0) | 10(6) | 4(4) |
| $\delta$ | 2(0) | 0(0) | 2(2) | 2(0) | 2(0) | 1(0) | 2(0) | 1(1) |
| $\theta$ | 2(1) | 3(2) | 2(1) | 1(0) | 1(0) | 1(1) | 2(1) | 0(0) |
| $\alpha_1$ | 0(0) | 2(1) | 11(6) | 1(1) | 1(1) | 0(0) | 1(0) | 1(1) |
| $\alpha_2$ | 0(0) | 1(1) | 3(0) | 0(0) | 0(0) | 0(0) | 0(0) | 1(0) |
| $\beta$ | 0(0) | 5(3) | 6(3) | 0(0) | 1(1) | 0(0) | 0(0) | 1(1) |
| $\gamma$ | 0(0) | 1(0) | 7(5) | 1(0) | 1(0) | 0(0) | 1(0) | 0(0) |

(d) $\mathcal{X}(\mathcal{N}) > \mathcal{X}(\mathcal{O})$

| | $BC$ | $c$ | $\mathcal{C}$ | $N$ | $E$ | $\beta_0$ | $\beta_1$ | $\beta_2$ |
|---|---|---|---|---|---|---|---|---|
| $B$ | 61(46) | 29(23) | 1(1) | 71(52) | 68(51) | 24(18) | 73(52) | 54(40) |
| $\delta$ | 45(33) | 6(5) | 0(0) | 57(41) | 54(38) | 0(0) | 56(41) | 10(5) |
| $\theta$ | 41(30) | 15(11) | 0(0) | 57(42) | 55(41) | 2(2) | 55(40) | 23(18) |
| $\alpha_1$ | 48(39) | 10(8) | 1(0) | 61(46) | 59(44) | 2(1) | 60(46) | 18(12) |
| $\alpha_2$ | 44(29) | 4(4) | 0(0) | 45(31) | 46(33) | 1(1) | 48(32) | 6(5) |
| $\beta$ | 53(37) | 13(9) | 0(0) | 56(36) | 56(37) | 2(1) | 56(38) | 20(12) |
| $\gamma$ | 37(24) | 4(2) | 0(0) | 44(32) | 46(31) | 1(1) | 44(30) | 4(2) |

Supplementary Table 3: Number of patients for whom there was a significant difference in the direction of the hypothesis of Figure 3 of the main text for each metric (columns) and frequency band (rows). For each case, we show the total number of patients as well as the number of SF patients, within parenthesis.

(a) $\mathcal{X}(\mathcal{RA}) > \mathcal{X}(\overline{\mathcal{RA}})$

| | $BC$ | $c$ | $\mathcal{C}$ | $N$ | $E$ | $\beta_0$ | $\beta_1$ | $\beta_2$ |
|---|---|---|---|---|---|---|---|---|
| $B$ | 0(0) | 2(0) | 3(3) | 5(3) | 5(3) | 0(0) | 7(5) | 3(2) |
| $\delta$ | 0(0) | 1(1) | 0(0) | 3(3) | 2(2) | 0(0) | 3(3) | 0(0) |
| $\theta$ | 0(0) | 1(1) | 7(5) | 3(2) | 2(1) | 0(0) | 2(1) | 1(1) |
| $\alpha_1$ | 0(0) | 0(0) | 1(1) | 6(3) | 5(3) | 1(1) | 4(3) | 0(0) |
| $\alpha_2$ | 1(0) | 2(1) | 2(2) | 3(1) | 5(2) | 1(1) | 6(2) | 2(1) |
| $\beta$ | 3(1) | 1(1) | 1(1) | 5(2) | 6(3) | 1(0) | 5(2) | 0(0) |
| $\gamma$ | 0(0) | 1(1) | 2(0) | 2(1) | 1(1) | 0(0) | 3(3) | 0(0) |

(b) $\mathcal{X}(\mathcal{RA}) > \mathcal{X}(\mathcal{O})$

| | $BC$ | $c$ | $\mathcal{C}$ | $N$ | $E$ | $\beta_0$ | $\beta_1$ | $\beta_2$ |
|---|---|---|---|---|---|---|---|---|
| $B$ | 0(0) | 2(0) | 11(8) | 5(3) | 5(3) | 0(0) | 7(5) | 4(2) |
| $\delta$ | 0(0) | 1(1) | 4(2) | 3(3) | 3(3) | 0(0) | 3(3) | 0(0) |
| $\theta$ | 0(0) | 2(1) | 9(7) | 1(0) | 1(0) | 0(0) | 1(0) | 0(0) |
| $\alpha_1$ | 0(0) | 1(0) | 6(4) | 6(3) | 5(3) | 1(1) | 4(3) | 1(0) |
| $\alpha_2$ | 1(0) | 3(1) | 6(5) | 2(1) | 3(2) | 1(1) | 4(2) | 3(2) |
| $\beta$ | 2(1) | 1(1) | 4(3) | 5(2) | 6(3) | 1(0) | 4(2) | 0(0) |
| $\gamma$ | 0(0) | 1(1) | 4(0) | 1(1) | 1(1) | 0(0) | 2(2) | 0(0) |

(c) $\mathcal{X}(\mathcal{RA}) > \mathcal{X}(\mathcal{N})$

| | $BC$ | $c$ | $\mathcal{C}$ | $N$ | $E$ | $\beta_0$ | $\beta_1$ | $\beta_2$ |
|---|---|---|---|---|---|---|---|---|
| $B$ | 15(7) | 4(3) | 2(1) | 14(10) | 9(6) | 3(2) | 12(9) | 7(4) |
| $\delta$ | 4(2) | 2(2) | 0(0) | 5(3) | 6(4) | 0(0) | 6(4) | 2(1) |
| $\theta$ | 7(3) | 1(1) | 4(2) | 5(4) | 4(4) | 0(0) | 4(3) | 3(3) |
| $\alpha_1$ | 10(7) | 3(3) | 0(0) | 10(7) | 10(6) | 1(0) | 8(5) | 2(2) |
| $\alpha_2$ | 5(1) | 1(0) | 3(2) | 5(0) | 5(1) | 0(0) | 9(3) | 1(0) |
| $\beta$ | 10(5) | 4(3) | 1(1) | 10(5) | 8(3) | 0(0) | 11(6) | 4(1) |
| $\gamma$ | 10(7) | 2(0) | 0(0) | 4(3) | 4(3) | 0(0) | 5(3) | 0(0) |

(d) $\mathcal{X}(\mathcal{N}) > \mathcal{X}(\mathcal{O})$

| | $BC$ | $c$ | $\mathcal{C}$ | $N$ | $E$ | $\beta_0$ | $\beta_1$ | $\beta_2$ |
|---|---|---|---|---|---|---|---|---|
| $B$ | 0(0) | 6(3) | 30(21) | 0(0) | 0(0) | 0(0) | 0(0) | 0(0) |
| $\delta$ | 0(0) | 3(3) | 25(21) | 0(0) | 0(0) | 0(0) | 0(0) | 0(0) |
| $\theta$ | 0(0) | 3(1) | 20(15) | 1(0) | 1(0) | 1(1) | 0(0) | 0(0) |
| $\alpha_1$ | 0(0) | 3(2) | 24(16) | 1(1) | 0(0) | 2(2) | 0(0) | 0(0) |
| $\alpha_2$ | 0(0) | 0(0) | 33(22) | 1(1) | 1(1) | 1(1) | 0(0) | 0(0) |
| $\beta$ | 0(0) | 1(1) | 32(18) | 0(0) | 0(0) | 0(0) | 0(0) | 0(0) |
| $\gamma$ | 0(0) | 4(4) | 27(16) | 0(0) | 0(0) | 0(0) | 0(0) | 0(0) |

Supplementary Table 4: Number of patients for whom there was a significant difference in opposite direction of the hypothesis of Figure 3 of the main text for each metric (columns) and frequency band (rows). For each case, we show the total number of patients as well as the number of SF patients, within parenthesis.

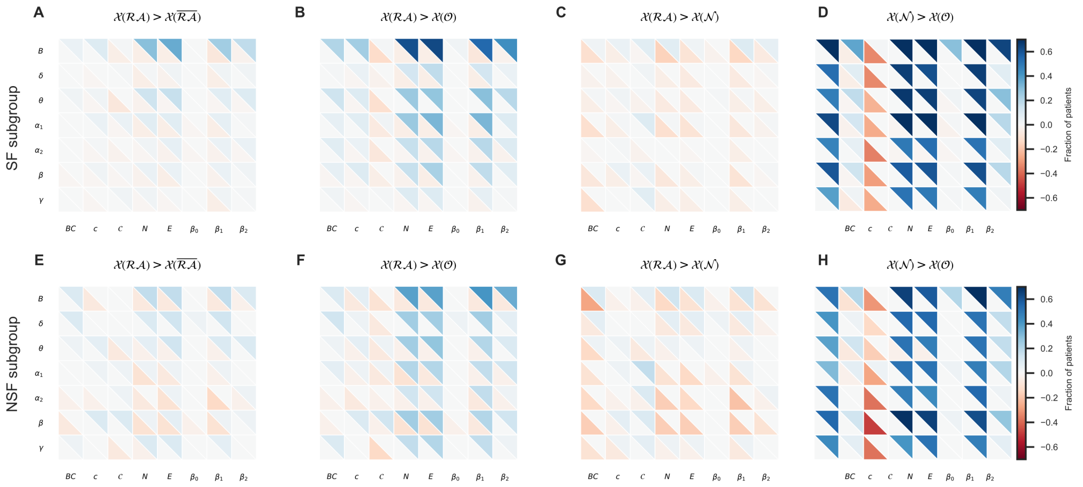


Supplementary Figure 3: Patient-specific comparison of different node groups for the two-group partition (panels A and F) and the three-group one (panels B, C, D, F, G, H), respectively for the SF (panels A-D) and NSF (panels E-H) subgroups. This supplementary figure repeats the analyses of Figure 3 of the main text, but separating the SF and NSF subgroups. For each panel, the hypothesis of the relation in centrality between the two compared node-groups is shown in the panel title. $\mathcal{X}(\mathcal{S})$ stands for the generalized centrality metric $\mathcal{X}$ measured on the nodes in set $\mathcal{S}$. The fraction of patients of each subgroup for whom there was a significant difference in the direction (opposite direction) of the hypothesis is shown by the blue (red) triangles in the upper-right (bottom-left) corner of each cell, respectively for each frequency band (rows) and generalized centrality metric (columns), color-coded as indicated by the color-bars. The corresponding numerical values for the SF group are shown in Supplementary Tables 3 and 4. Numerical values for the NSF can be directly derived by subtracting the SF values from those of the full cohort, reported also in the same tables. Significance (threshold $= 0.05$) was established with a bootstrapping analysis ($n = 10^4$, see Methods). The number of samples in each test was different for each patient since it was given by the size of each node-set. The $p$-values were Bonferroni corrected for multiple comparisons ($n = 56$).

| Comparison | Direction | SF | NSF | Diff. | $p$ | $r$ |
|---|---|---|---|---|---|---|
| $\mathcal{X}(\mathcal{RA}) > \mathcal{X}(\overline{\mathcal{RA}})$ | Positive | 0.051 | 0.048 | 0.003 | 0.732 | 8.125 |
| | Negative | 0.029 | 0.068 | −0.008 | 0.091 | 5.071 |
| $\mathcal{X}(\mathcal{RA}) > \mathcal{X}(\mathcal{O})$ | Positive | 0.143 | 0.126 | 0.017 | 0.238 | 9.268 |
| | Negative | 0.025 | 0.034 | −0.009 | 0.075 (*) | 5.375 |
| $\mathcal{X}(\mathcal{RA}) > \mathcal{X}(\mathcal{N})$ | Positive | 0.013 | 0.025 | −0.013 | 0.003 (**) | 2.161 |
| | Negative | 0.045 | 0.068 | −0.023 | 0.034 (**) | 5.875 |
| $\mathcal{X}(\mathcal{N}) > \mathcal{X}(\mathcal{O})$ | Positive | 0.398 | 0.341 | 0.057 | 0.003 (**) | 6.429 |
| | Negative | 0.043 | 0.03 | 0.000 | 0.931 | 2.018 |

Supplementary Table 5: Comparison of the fraction of patients for whom there was a significant difference in the direction of the hypothesis (direction = positive), or against it (direction = negative), for the SF and NSF groups. We report the results for each type of comparison independently, and averaged over frequency bands and metrics. Given that the curvature changes in opposite direction to the other metrics (i.e. more negative values are associated with hubness), the directions have been inverted for this metric. We include the comparison between the SF and NSF subgroups by indicating the difference (diff.), FDR-corrected $p$-value, and $r$-value according the Wilcoxon signed-rank test. Significant differences are indicated by a double asterisk next to the $p$-value (threshold = 0.05), and those that did not survive FDR correction are indicated with a single asterisk.

## S.6. SUPPLEMENTARY INFORMATION TO THE GROUP-LEVEL COMPARISONS

In this section we provide supplementary information to the analyses presented in Figure 4 of the main text. Firstly, in Supplementary Tables 6 and 7 we show the z-scores and corresponding $p$-values.

In order to identify whether the results hold for the SF and NSF subgroups independently, we repeated the analyses in Figure 4 for each subgroup. The results are shown in Supplementary Figure 4, with supporting numerical data reported in Supplementary Tables 8, 10 ($z$-scores of the difference), 9, and 11 ($p$-values) respectively for the SF and NSF subgroups.

(a) $\mathcal{X}(\mathcal{RA}) > \mathcal{X}(\overline{\mathcal{RA}})$

| | $BC$ | $c$ | $\mathcal{C}$ | $N$ | $E$ | $\beta_0$ | $\beta_1$ | $\beta_2$ |
|---|---|---|---|---|---|---|---|---|
| $B$ | 2.2 | 1.5 | -1.7 | 4.2 | 3.8 | 2.7 | 2.0 | 1.4 |
| $\delta$ | 0.5 | 0.3 | 0.6 | 0.6 | 0.5 | 0.5 | 0.4 | 0.2 |
| $\theta$ | 0.9 | 1.8 | -0.5 | 3.1 | 3.2 | -0.1 | 1.2 | 0.9 |
| $\alpha_1$ | -2.1 | 0.5 | 0.8 | 0.3 | 0.7 | -2.2 | 0.6 | 0.5 |
| $\alpha_2$ | -0.9 | -1.0 | -1.2 | 0.5 | 0.5 | -0.8 | -0.4 | -0.1 |
| $\beta$ | -1.2 | 1.0 | 0.4 | 0.4 | 0.4 | -1.1 | 0.5 | 0.6 |
| $\gamma$ | -2.1 | -1.8 | -0.4 | -0.7 | -0.6 | -1.4 | 0.3 | 0.4 |

(b) $\mathcal{X}(\mathcal{RA}) > \mathcal{X}(\mathcal{O})$

| | $BC$ | $c$ | $\mathcal{C}$ | $N$ | $E$ | $\beta_0$ | $\beta_1$ | $\beta_2$ |
|---|---|---|---|---|---|---|---|---|
| $B$ | -4.2 | 0.1 | 1.6 | -1.9 | -1.4 | -3.3 | -1.2 | -0.4 |
| $\delta$ | -3.2 | 0.3 | 3.5 | -3.6 | -3.0 | 0.3 | -2.9 | -0.4 |
| $\theta$ | -3.6 | 1.0 | 1.2 | -2.5 | -1.7 | -0.7 | -1.5 | 0.2 |
| $\alpha_1$ | -6.5 | 0.9 | 2.7 | -2.9 | -2.3 | -1.9 | -1.9 | -0.2 |
| $\alpha_2$ | -5.6 | 0.5 | 0.5 | -1.1 | -2.3 | -2.3 | -0.04 | 0.0 |
| $\beta$ | -7.5 | 0.5 | 2.8 | -4.1 | -3.5 | -1.5 | -4.1 | -0.4 |
| $\gamma$ | -6.8 | -1.0 | -3.7 | -4.2 | -4.1 | -1.8 | -0.2 | 0.05 |

(c) $\mathcal{X}(\mathcal{RA}) > \mathcal{X}(\mathcal{N})$

| | $BC$ | $c$ | $\mathcal{C}$ | $N$ | $E$ | $\beta_0$ | $\beta_1$ | $\beta_2$ |
|---|---|---|---|---|---|---|---|---|
| $B$ | 5.7 | 3.4 | -3.7 | 8.1 | 7.7 | 5.4 | 4.4 | 2.9 |
| $\delta$ | 3.4 | 0.7 | -1.6 | 4.0 | 3.6 | 0.6 | 0.8 | 0.9 |
| $\theta$ | 3.9 | 2.6 | -2.1 | 6.1 | 6.5 | 0.5 | 3.2 | 2.2 |
| $\alpha_1$ | 2.1 | 0.8 | -0.5 | 4.8 | 3.5 | 0.7 | 0.4 | 1.0 |
| $\alpha_2$ | -0.6 | -0.9 | -0.5 | 4.7 | 3.1 | -0.5 | 2.0 | 0.09 |
| $\beta$ | 3.1 | -1.5 | -1.5 | 3.5 | 3.5 | -0.7 | 1.2 | 1.4 |
| $\gamma$ | 1.6 | -2.1 | -2.1 | 3.5 | 3.1 | -0.1 | 1.3 | 0.6 |

(d) $\mathcal{X}(\mathcal{N}) > \mathcal{X}(\mathcal{O})$

| | $BC$ | $c$ | $\mathcal{C}$ | $N$ | $E$ | $\beta_0$ | $\beta_1$ | $\beta_2$ |
|---|---|---|---|---|---|---|---|---|
| $B$ | 15 | 4.1 | -5.7 | 13 | 12 | 8.0 | 6.2 | 1.2 |
| $\delta$ | 17 | 0.7 | -7.0 | 11 | 8.1 | 7.9 | 7.1 | 1.2 |
| $\theta$ | 7.5 | 2.1 | -3.6 | 11 | 11 | 1.5 | 5.2 | 2.2 |
| $\alpha_1$ | 10 | -0.3 | -4.8 | 11 | 8.3 | 5.6 | 1.2 | 1.0 |
| $\alpha_2$ | 15 | -0.3 | -4.7 | 7.0 | 7.0 | -0.5 | 5.5 | 0.06 |
| $\beta$ | 18 | 1.3 | -5.7 | 13 | 10 | 1.8 | 7.3 | 1.8 |
| $\gamma$ | 13 | -1.5 | -6.5 | 8.5 | 7.9 | 1.4 | 8.0 | 0.6 |

Supplementary Table 6: Numerical values corresponding to results of Figure 4 of the main text. Group-level comparison between nodes sets, for each considered frequency-band (y-axis) and network metric (x-axis). $\mathcal{X}(S)$ stands for the generalized centrality metric $\mathcal{X}$ measured on the nodes in set $S$. The numbers indicate the $z$-score of the difference between the average values of each node set, computed by bootstrapping the data (sampling size of $10^4$).

(a) $\mathcal{X}(\mathcal{RA}) > \mathcal{X}(\overline{\mathcal{RA}})$

| | $BC$ | $c$ | $\mathcal{C}$ | $N$ | $E$ | $\beta_0$ | $\beta_1$ | $\beta_2$ |
|---|---|---|---|---|---|---|---|---|
| $B$ | 0.03 | 0.1 | 0.08 | $< 10^{-3}$ | $< 10^{-3}$ | 0.007 | 0.05 | 0.2 |
| $\delta$ | 0.6 | 0.8 | 0.5 | 0.6 | 0.6 | 0.6 | 0.7 | 0.9 |
| $\theta$ | 0.4 | 0.07 | 0.6 | 0.002 | 0.002 | 0.9 | 0.2 | 0.2 |
| $\alpha_1$ | 0.04 | 0.6 | 0.4 | 0.8 | 0.5 | 0.03 | 0.6 | 0.6 |
| $\alpha_2$ | 0.4 | 0.3 | 0.2 | 0.6 | 0.6 | 0.5 | 0.7 | 0.9 |
| $\beta$ | 0.2 | 0.3 | 0.7 | 0.7 | 0.7 | 0.3 | 0.6 | 0.6 |
| $\gamma$ | 0.04 | 0.07 | 0.7 | 0.5 | 0.5 | 0.8 | 0.5 | 0.7 |

(b) $\mathcal{X}(\mathcal{RA}) > \mathcal{X}(\mathcal{O})$

| | $BC$ | $c$ | $\mathcal{C}$ | $N$ | $E$ | $\beta_0$ | $\beta_1$ | $\beta_2$ |
|---|---|---|---|---|---|---|---|---|
| $B$ | $< 10^{-3}$ | 0.9 | 0.1 | 0.06 | 0.2 | $10^{-3}$ | 0.2 | 0.7 |
| $\delta$ | $10^{-3}$ | 0.8 | $10^{-3}$ | $< 10^{-3}$ | 0.003 | 0.8 | 0.003 | 0.7 |
| $\theta$ | $< 10^{-3}$ | 0.3 | 0.2 | 0.01 | 0.09 | 0.5 | 0.1 | 0.9 |
| $\alpha_1$ | $< 10^{-3}$ | 0.4 | 0.008 | 0.003 | 0.01 | 0.05 | 0.06 | 0.8 |
| $\alpha_2$ | $< 10^{-3}$ | 0.5 | 0.3 | 0.02 | 0.02 | 0.9 | 0.05 | 0.9 |
| $\beta$ | $< 10^{-3}$ | 0.5 | 0.005 | $< 10^{-3}$ | $< 10^{-3}$ | 0.1 | $< 10^{-3}$ | 0.7 |
| $\gamma$ | $< 10^{-3}$ | 0.3 | $< 10^{-3}$ | $< 10^{-3}$ | $< 10^{-3}$ | 0.5 | $< 10^{-3}$ | 0.9 |

Supplementary Table 7: Continued in next page.

(c) $\mathcal{X}(\mathcal{RA}) > \mathcal{X}(\mathcal{N})$

| | $BC$ | $c$ | $\mathcal{C}$ | $N$ | $E$ | $\beta_0$ | $\beta_1$ | $\beta_2$ |
|---|---|---|---|---|---|---|---|---|
| $B$ | $< 10^{-3}$ | $< 0^{-3}$ | $< 10^{-3}$ | $< 10^{-3}$ | $< 10^{-3}$ | $< 10^{-3}$ | $< 10^{-3}$ | 0.004 |
| $\delta$ | $10^{-3}$ | 0.5 | 0.1 | $< 10^{-3}$ | $< 10^{-3}$ | 0.5 | 0.002 | 0.4 |
| $\theta$ | $< 10^{-3}$ | 0.01 | 0.08 | $< 10^{-3}$ | $< 10^{-3}$ | 0.6 | 0.001 | 0.03 |
| $\alpha_1$ | 0.03 | 0.4 | 0.6 | 0.003 | $< 10^{-3}$ | 0.04 | 0.006 | 0.3 |
| $\alpha_2$ | 0.001 | 0.4 | 0.003 | 0.002 | 0.002 | 0.6 | 0.01 | 0.9 |
| $\beta$ | 0.002 | 0.1 | 0.1 | $< 10^{-3}$ | $< 10^{-3}$ | 0.5 | 0.003 | 0.2 |
| $\gamma$ | 0.1 | 0.04 | 0.04 | 0.07 | 0.06 | 0.9 | 0.2 | 0.6 |

(d) $\mathcal{X}(\mathcal{N}) > \mathcal{X}(\mathcal{O})$

| | $BC$ | $c$ | $\mathcal{C}$ | $N$ | $E$ | $\beta_0$ | $\beta_1$ | $\beta_2$ |
|---|---|---|---|---|---|---|---|---|
| $B$ | $< 10^{-3}$ | $< 10^{-3}$ | $< 10^{-3}$ | $< 10^{-3}$ | $< 10^{-3}$ | $< 10^{-3}$ | $< 10^{-3}$ | $< 10^{-3}$ |
| $\delta$ | $< 10^{-3}$ | 0.5 | $< 10^{-3}$ | $< 10^{-3}$ | 0.3 | $< 10^{-3}$ | $< 10^{-3}$ | 0.224 |
| $\theta$ | $< 10^{-3}$ | 0.04 | $< 10^{-3}$ | $< 10^{-3}$ | 0.1 | $< 10^{-3}$ | $< 10^{-3}$ | 0.025 |
| $\alpha_1$ | $< 10^{-3}$ | 0.8 | $< 10^{-3}$ | $< 10^{-3}$ | 0.4 | $< 10^{-3}$ | $< 10^{-3}$ | 0.248 |
| $\alpha_2$ | $< 10^{-3}$ | 0.8 | $< 10^{-3}$ | $< 10^{-3}$ | 0.6 | $< 10^{-3}$ | $< 10^{-3}$ | 0.955 |
| $\beta$ | $< 10^{-3}$ | 0.2 | $< 10^{-3}$ | $< 10^{-3}$ | 0.07 | $< 10^{-3}$ | $< 10^{-3}$ | 0.071 |
| $\gamma$ | $< 10^{-3}$ | 0.1 | $< 10^{-3}$ | $< 10^{-3}$ | 0.5 | $< 10^{-3}$ | $< 10^{-3}$ | 0.531 |

Supplementary Table 7: p-values corresponding to results of Figure 4 of the main text. Group-level comparison between nodes sets, for each considered frequency-band (y-axis) and network metric (x-axis). $\mathcal{X}(S)$ stands for the generalized centrality metric $\mathcal{X}$ measured on the nodes in set $S$. The numbers indicate the p-values of the comparison between the average values of each node set presented in Supplementary Table 6 and in Figure 4 of the main text.

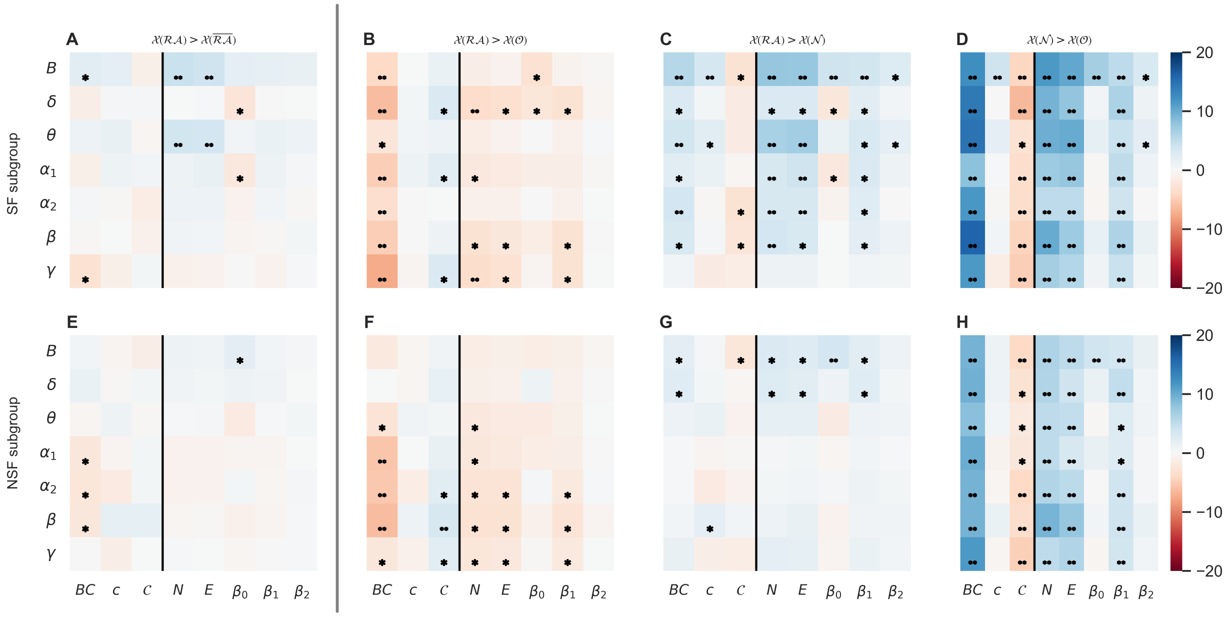


Supplementary Figure 4: Group-level comparison between node-sets, for each considered frequency-band (y-axis) and network metric (x-axis), independently for the SF (panels A-D) and NSF (panels E-H) subgroups. This supplementary figure repeats the analyses on Figure 4 of the main text but independently for the SF and NSF subgroups. From left to right, the panels indicate the difference between the node sets: i) $\mathcal{RA}$ vs $\overline{\mathcal{RA}}$ (panels A and E), ii) $\mathcal{RA}$ vs $\mathcal{N}$ (panels B and F), iii) $\mathcal{RA}$ vs $\mathcal{O}$ (panels C and G), iv) $\mathcal{N}$ vs $\mathcal{O}$ (panels D and H). $\mathcal{X}(S)$ stands for the generalized centrality metric $\mathcal{X}$ measured on the nodes in set $S$. The color code indicates the $z$-score of the difference between the average values of each node set, computed by bootstrapping the data (sampling size of $10^4$). The $p$-values were Bonferroni corrected for multiple comparisons ($n = 56$). Double asterisks indicate significant differences, and single asterisks those that did not survive the correction. Significance (threshold $= 0.05$) was established with a paired bootstrapping analysis ($n = 10^4$, see Methods). The number of paired samples in each test was equal to the number of patients, $N = 91$.

(a) $\mathcal{X}(\mathcal{RA}) > \mathcal{X}(\overline{\mathcal{RA}})$

| | $BC$ | $c$ | $\mathcal{C}$ | $N$ | $E$ | $\beta_0$ | $\beta_1$ | $\beta_2$ |
|---|---|---|---|---|---|---|---|---|
| $B$ | 2.3 | 1.9 | −1.2 | 4.3 | 4.1 | 2.0 | 1.8 | 1.4 |
| $\delta$ | −1.4 | 0.4 | 0.4 | 0.1 | 0.2 | −2.5 | −0.07 | 0.1 |
| $\theta$ | 1.2 | 1.6 | −0.4 | 3.8 | 3.6 | 0.7 | 1.4 | 1.1 |
| $\alpha_1$ | −1.2 | 1.0 | 0.6 | 1.0 | 1.5 | −2.2 | 1.0 | 0.5 |
| $\alpha_2$ | 0.4 | −0.3 | −1.6 | 1.1 | 1.1 | −0.9 | 0.8 | −0.3 |
| $\beta$ | −0.2 | 0.1 | −1.0 | 0.9 | 0.7 | −0.5 | −0.2 | 0.6 |
| $\gamma$ | −3.1 | −1.3 | 0.5 | −1.0 | −0.8 | −0.09 | −0.8 | 0.3 |

(b) $\mathcal{X}(\mathcal{RA}) > \mathcal{X}(\mathcal{O})$

| | $BC$ | $c$ | $\mathcal{C}$ | $N$ | $E$ | $\beta_0$ | $\beta_1$ | $\beta_2$ |
|---|---|---|---|---|---|---|---|---|
| $B$ | −3.9 | 0.08 | 1.3 | −1.3 | −0.9 | −3.0 | −0.7 | −0.2 |
| $\delta$ | −6.3 | 0.6 | 3.0 | −3.6 | −3.2 | −2.5 | −2.9 | −0.4 |
| $\theta$ | −2.6 | 0.7 | 1.1 | −1.5 | −0.9 | −0.01 | −1.0 | 0.2 |
| $\alpha_1$ | −4.9 | 1.1 | 2.2 | −2.0 | −1.8 | −1.3 | −1.4 | −0.2 |
| $\alpha_2$ | −3.4 | −0.09 | 0.08 | −1.1 | −1.0 | −0.1 | −1.1 | 0.03 |
| $\beta$ | −5.0 | −0.07 | 1.0 | −2.8 | −2.2 | −1.4 | −3.0 | −0.1 |
| $\gamma$ | −7.3 | −0.5 | 2.9 | −3.5 | −3.1 | −0.6 | −2.9 | 0.01 |

Supplementary Table 8: Continued in next page.

(c) $\mathcal{X}(\mathcal{RA}) > \mathcal{X}(\mathcal{N})$

| | $BC$ | $c$ | $\mathcal{C}$ | $N$ | $E$ | $\beta_0$ | $\beta_1$ | $\beta_2$ |
|---|---|---|---|---|---|---|---|---|
| $B$ | 5.7 | 3.7 | −2.8 | 7.7 | 7.5 | 4.1 | 3.9 | 2.6 |
| $\delta$ | 2.9 | 0.6 | −1.7 | 3.0 | 2.9 | −2.2 | 2.2 | 0.6 |
| $\theta$ | 3.9 | 2.2 | −1.5 | 6.4 | 6.8 | 1.3 | 3.2 | 2.0 |
| $\alpha_1$ | 2.3 | 1.3 | −0.4 | 3.4 | 4.1 | −2.1 | 2.9 | 1.0 |
| $\alpha_2$ | 3.7 | −0.2 | −2.9 | 3.3 | 3.3 | −0.6 | 2.4 | −0.06 |
| $\beta$ | 3.2 | 0.4 | −2.6 | 3.6 | 3.2 | 0.06 | 2.1 | 1.2 |
| $\gamma$ | 0.7 | −1.6 | −1.5 | 1.0 | 1.1 | 0.09 | 0.8 | 0.5 |

(d) $\mathcal{X}(\mathcal{N}) > \mathcal{X}(\mathcal{O})$

| | $BC$ | $c$ | $\mathcal{C}$ | $N$ | $E$ | $\beta_0$ | $\beta_1$ | $\beta_2$ |
|---|---|---|---|---|---|---|---|---|
| $B$ | 12 | 4.2 | −4.2 | 11 | 10.4 | 6.8 | 5.2 | 3.2 |
| $\delta$ | 14 | −0.03 | −6.4 | 9.6 | 8.0 | 0.8 | 6.2 | 1.0 |
| $\theta$ | 15 | 2.0 | −3.0 | 9.7 | 10.2 | 1.8 | 4.4 | 2.1 |
| $\alpha_1$ | 8.0 | 0.3 | −3.5 | 7.4 | 7.7 | −0.2 | 5.1 | 1.1 |
| $\alpha_2$ | 12 | −0.2 | −3.4 | 5.2 | 5.2 | −0.5 | 4.1 | −0.08 |
| $\beta$ | 16 | 0.7 | −4.5 | 10.0 | 7.3 | 1.7 | 6.1 | 1.3 |
| $\gamma$ | 12 | −1.5 | −4.9 | 7.0 | 5.8 | 0.9 | 4.4 | 0.47 |

Supplementary Table 8: Numerical values of the $z$-scores of the average difference between node-sets corresponding to the results of panels A-D of Supplementary Figure 4 corresponding to the SF subgroup. The $z$-scores were computed by bootstrapping the data (sampling size of $10^4$). Group-level comparison between nodes sets, for each considered frequency-band (y-axis) and network metric (x-axis). $\mathcal{X}(S)$ stands for the generalized centrality metric $\mathcal{X}$ measured on the nodes in set $S$.

(a) $\mathcal{X}(\mathcal{RA}) > \mathcal{X}(\overline{\mathcal{RA}})$

| | $BC$ | $c$ | $\mathcal{C}$ | $N$ | $E$ | $\beta_0$ | $\beta_1$ | $\beta_2$ |
|---|---|---|---|---|---|---|---|---|
| $B$ | 0.03 | 0.06 | 0.2 | $< 10^{-3}$ | $< 10^{-3}$ | 0.05 | 0.07 | 0.2 |
| $\delta$ | 0.2 | 0.7 | 0.7 | 0.9 | 0.9 | 0.01 | 0.9 | 0.9 |
| $\theta$ | 0.2 | 0.1 | 0.7 | $< 10^{-3}$ | $< 10^{-3}$ | 0.5 | 0.2 | 0.2 |
| $\alpha_1$ | 0.2 | 0.3 | 0.5 | 0.3 | 0.1 | 0.03 | 0.3 | 0.7 |
| $\alpha_2$ | 0.7 | 0.8 | 0.1 | 0.3 | 0.3 | 0.4 | 0.4 | 0.8 |
| $\beta$ | 0.8 | 0.9 | 0.3 | 0.4 | 0.5 | 0.6 | 0.9 | 0.5 |
| $\gamma$ | 0.002 | 0.2 | 0.6 | 0.3 | 0.4 | 0.9 | 0.4 | 0.8 |

(b) $\mathcal{X}(\mathcal{RA}) > \mathcal{X}(\mathcal{O})$

| | $BC$ | $c$ | $\mathcal{C}$ | $N$ | $E$ | $\beta_0$ | $\beta_1$ | $\beta_2$ |
|---|---|---|---|---|---|---|---|---|
| $B$ | $< 10^{-3}$ | 0.9 | 0.2 | 0.2 | 0.4 | 0.003 | 0.5 | 0.8 |
| $\delta$ | $< 10^{-3}$ | 0.6 | 0.002 | $< 10^{-3}$ | 0.001 | 0.01 | 0.004 | 0.7 |
| $\theta$ | 0.008 | 0.5 | 0.3 | 0.1 | 0.4 | 1.0 | 0.3 | 0.9 |
| $\alpha_1$ | $< 10^{-3}$ | 0.3 | 0.03 | 0.04 | 0.1 | 0.2 | 0.2 | 0.8 |
| $\alpha_2$ | $< 10^{-3}$ | 0.9 | 0.9 | 0.3 | 0.3 | 0.9 | 0.3 | 1.0 |
| $\beta$ | $< 10^{-3}$ | 0.9 | 0.3 | 0.005 | 0.03 | 0.2 | 0.003 | 0.9 |
| $\gamma$ | $< 10^{-3}$ | 0.6 | 0.004 | $< 10^{-3}$ | 0.002 | 0.5 | 0.003 | 1.0 |

Supplementary Table 9: Continued in next page.

(c) $\mathcal{X}(\mathcal{RA}) > \mathcal{X}(\mathcal{N})$

| | $BC$ | $c$ | $\mathcal{C}$ | $N$ | $E$ | $\beta_0$ | $\beta_1$ | $\beta_2$ |
|---|---|---|---|---|---|---|---|---|
| $B$ | $< 10^{-3}$ | $< 10^{-3}$ | 0.005 | $< 10^{-3}$ | $< 10^{-3}$ | $< 10^{-3}$ | $< 10^{-3}$ | 0.006 |
| $\delta$ | 0.004 | 0.6 | 0.1 | 0.003 | 0.004 | 0.03 | 0.03 | 0.5 |
| $\theta$ | $< 10^{-3}$ | 0.02 | 0.1 | $< 10^{-3}$ | $< 10^{-3}$ | 0.2 | 0.002 | 0.05 |
| $\alpha_1$ | 0.02 | 0.2 | 0.6 | $< 10^{-3}$ | $< 10^{-3}$ | 0.04 | 0.004 | 0.3 |
| $\alpha_2$ | $< 10^{-3}$ | 0.8 | 0.003 | $< 10^{-3}$ | $< 10^{-3}$ | 0.5 | 0.02 | 1.0 |
| $\beta$ | 0.002 | 0.7 | 0.009 | $< 10^{-3}$ | 0.001 | 0.9 | 0.03 | 0.2 |
| $\gamma$ | 0.5 | 0.1 | 0.1 | 0.3 | 0.3 | 0.9 | 0.4 | 0.6 |

(d) $\mathcal{X}(\mathcal{N}) > \mathcal{X}(\mathcal{O})$

| | $BC$ | $c$ | $\mathcal{C}$ | $N$ | $E$ | $\beta_0$ | $\beta_1$ | $\beta_2$ |
|---|---|---|---|---|---|---|---|---|
| $B$ | $< 10^{-3}$ | $< 10^{-3}$ | $< 10^{-3}$ | $< 10^{-3}$ | $< 10^{-3}$ | $< 10^{-3}$ | $< 10^{-3}$ | 0.002 |
| $\delta$ | $< 10^{-3}$ | 0.9 | $< 10^{-3}$ | $< 10^{-3}$ | $< 10^{-3}$ | 0.4 | $< 10^{-3}$ | 0.3 |
| $\theta$ | $< 10^{-3}$ | 0.05 | 0.002 | $< 10^{-3}$ | $< 10^{-3}$ | 0.07 | $< 10^{-3}$ | 0.04 |
| $\alpha_1$ | $< 10^{-3}$ | 0.8 | $< 10^{-3}$ | $< 10^{-3}$ | $< 10^{-3}$ | 0.9 | $< 10^{-3}$ | 0.2 |
| $\alpha_2$ | $< 10^{-3}$ | 0.9 | 0.001 | $< 10^{-3}$ | $< 10^{-3}$ | 0.6 | $< 10^{-3}$ | 0.9 |
| $\beta$ | $< 10^{-3}$ | 0.5 | $< 10^{-3}$ | $< 10^{-3}$ | $< 10^{-3}$ | 0.09 | $< 10^{-3}$ | 0.2 |
| $\gamma$ | $< 10^{-3}$ | 0.1 | $< 10^{-3}$ | $< 10^{-3}$ | $< 10^{-3}$ | 0.4 | $< 10^{-3}$ | 0.6 |

Supplementary Table 9: Numerical $p$-values corresponding to the results of panels A-D of Supplementary Figure 4 for the SF group. These correspond to the group-level comparison between nodes sets, for each considered frequency-band (y-axis) and network metric (x-axis). $\mathcal{X}(S)$ stands for the generalized centrality metric $\mathcal{X}$ measured on the nodes in set $S$.

(a) $\mathcal{X}(\mathcal{RA}) > \mathcal{X}(\overline{\mathcal{RA}})$

| | $BC$ | $c$ | $\mathcal{C}$ | $N$ | $E$ | $\beta_0$ | $\beta_1$ | $\beta_2$ |
|---|---|---|---|---|---|---|---|---|
| $B$ | 0.8 | −0.5 | −1.4 | 1.1 | 0.9 | 2.1 | 0.8 | 0.3 |
| $\delta$ | 1.4 | −0.3 | 0.5 | 0.8 | 0.5 | 1.1 | 0.9 | 0.2 |
| $\theta$ | −0.3 | 1.0 | −0.3 | 0.3 | 0.4 | −1.9 | 0.2 | 0.5 |
| $\alpha_1$ | −2.2 | −0.6 | 0.5 | −1.0 | −0.8 | −0.6 | −0.5 | 0.1 |
| $\alpha_2$ | −2.5 | −1.7 | 0.5 | −0.8 | −0.8 | 0.5 | −0.4 | 0.4 |
| $\beta$ | −2.3 | 1.7 | 1.8 | −0.4 | −0.3 | −1.0 | −0.6 | 0.2 |
| $\gamma$ | −0.1 | −1.4 | 0.02 | 0.2 | 0.1 | −0.4 | −0.02 | 0.3 |

(b) $\mathcal{X}(\mathcal{RA}) > \mathcal{X}(\mathcal{O})$

| | $BC$ | $c$ | $\mathcal{C}$ | $N$ | $E$ | $\beta_0$ | $\beta_1$ | $\beta_2$ |
|---|---|---|---|---|---|---|---|---|
| $B$ | −1.9 | −0.4 | 0.8 | −1.4 | −1.2 | −1.5 | −1.2 | −0.4 |
| $\delta$ | 0.1 | −0.5 | 1.7 | −1.2 | −1.0 | 1.0 | −1.0 | −0.1 |
| $\theta$ | −2.8 | 0.8 | 0.6 | −2.1 | −1.8 | −1.9 | −1.2 | $< 10^{-3}$ |
| $\alpha_1$ | −5.5 | 0.07 | 1.5 | −2.3 | −1.9 | −1.5 | −1.4 | −0.06 |
| $\alpha_2$ | −5.3 | −1.4 | 2.4 | −2.7 | −2.8 | 0.5 | −2.0 | 0.2 |
| $\beta$ | −6.3 | 1.2 | 3.3 | −3.1 | −3.0 | −1.1 | −2.8 | −0.6 |
| $\gamma$ | −2.3 | −1.07 | 2.2 | −2.4 | −2.1 | −0.2 | −2.0 | 0.04 |

Supplementary Table 10: Continued next page.

(c) $\mathcal{X}(\mathcal{RA}) > \mathcal{X}(\mathcal{N})$

| | $BC$ | $c$ | $\mathcal{C}$ | $N$ | $E$ | $\beta_0$ | $\beta_1$ | $\beta_2$ |
|---|---|---|---|---|---|---|---|---|
| $B$ | 2.2 | 0.4 | −2.4 | 3.1 | 2.7 | 3.7 | 2.2 | 1.2 |
| $\delta$ | 2.3 | 0.5 | −0.4 | 2.7 | 2.2 | 1.2 | 2.5 | 0.6 |
| $\theta$ | 1.2 | 1.3 | −0.9 | 1.8 | 1.9 | −1.7 | 1.1 | 0.9 |
| $\alpha_1$ | 0.2 | −0.5 | −0.2 | 0.4 | 0.5 | −0.5 | 0.6 | 0.3 |
| $\alpha_2$ | 0.3 | −1.8 | −0.9 | 0.7 | 0.7 | 0.4 | 1.0 | 0.5 |
| $\beta$ | 0.7 | 2.0 | 0.7 | 1.2 | 1.5 | −0.9 | 0.8 | 0.8 |
| $\gamma$ | 1.5 | −1.3 | −1.5 | 1.8 | 1.8 | −0.6 | 1.3 | 0.7 |

(d) $\mathcal{X}(\mathcal{N}) > \mathcal{X}(\mathcal{O})$

| | $BC$ | $c$ | $\mathcal{C}$ | $N$ | $E$ | $\beta_0$ | $\beta_1$ | $\beta_2$ |
|---|---|---|---|---|---|---|---|---|
| $B$ | 9.4 | 0.9 | −4.0 | 6.1 | 5.2 | 4.0 | 3.6 | 1.6 |
| $\delta$ | 9.5 | 1.2 | −2.8 | 5.9 | 4.2 | 1.3 | 5.1 | 0.7 |
| $\theta$ | 8.3 | 0.7 | −2.0 | 5.2 | 5.0 | −0.3 | 2.7 | 0.9 |
| $\alpha_1$ | 10.0 | −0.8 | −2.3 | 5.1 | 3.4 | 1.4 | 2.5 | 0.3 |
| $\alpha_2$ | 9.4 | −0.4 | −4.0 | 6.1 | 5.4 | 0.0 | 3.9 | 0.4 |
| $\beta$ | 9.6 | 1.3 | −3.5 | 9.3 | 7.7 | 0.7 | 4.1 | 1.4 |
| $\gamma$ | 11 | −0.3 | −4.8 | 5.1 | 6.0 | −0.4 | 3.8 | 0.6 |

Supplementary Table 10: Numerical values of the $z$-scores of the average difference between node-sets corresponding to the results of panels E-H of Supplementary Figure 4, corresponding to the NSF subgroup. The $z$-scores were computed by bootstrapping the data (sampling size of $10^4$). Group-level comparison between nodes sets, for each considered frequency-band (y-axis) and network metric (x-axis). $\mathcal{X}(S)$ stands for the generalized centrality metric $\mathcal{X}$ measured on the nodes in set $S$.

(a) $\mathcal{X}(\mathcal{RA}) > \mathcal{X}(\overline{\mathcal{RA}})$

| | $BC$ | $c$ | $\mathcal{C}$ | $N$ | $E$ | $\beta_0$ | $\beta_1$ | $\beta_2$ |
|---|---|---|---|---|---|---|---|---|
| $B$ | 0.4 | 0.6 | 0.2 | 0.3 | 0.4 | 0.03 | 0.5 | 0.8 |
| $\delta$ | 0.2 | 0.8 | 0.6 | 0.4 | 0.6 | 0.3 | 0.4 | 0.9 |
| $\theta$ | 0.8 | 0.3 | 0.8 | 0.8 | 0.7 | 0.06 | 0.8 | 0.6 |
| $\alpha_1$ | 0.03 | 0.6 | 0.6 | 0.3 | 0.4 | 0.6 | 0.6 | 0.9 |
| $\alpha_2$ | 0.01 | 0.09 | 0.6 | 0.4 | 0.4 | 0.6 | 0.7 | 0.7 |
| $\beta$ | 0.02 | 0.09 | 0.07 | 0.7 | 0.8 | 0.3 | 0.5 | 0.9 |
| $\gamma$ | 0.9 | 0.2 | 1.0 | 0.9 | 0.9 | 0.7 | 1.0 | 0.8 |

(b) $\mathcal{X}(\mathcal{RA}) > \mathcal{X}(\mathcal{O})$

| | $BC$ | $c$ | $\mathcal{C}$ | $N$ | $E$ | $\beta_0$ | $\beta_1$ | $\beta_2$ |
|---|---|---|---|---|---|---|---|---|
| $B$ | 0.06 | 0.7 | 0.4 | 0.2 | 0.2 | 0.1 | 0.2 | 0.7 |
| $\delta$ | 0.9 | 0.6 | 0.09 | 0.2 | 0.3 | 0.3 | 0.3 | 0.9 |
| $\theta$ | 0.005 | 0.4 | 0.5 | 0.04 | 0.07 | 0.06 | 0.2 | 1.0 |
| $\alpha_1$ | $< 10^{-3}$ | 0.9 | 0.1 | 0.02 | 0.05 | 0.1 | 0.2 | 1.0 |
| $\alpha_2$ | $< 10^{-3}$ | 0.2 | 0.02 | 0.007 | 0.005 | 0.6 | 0.04 | 0.9 |
| $\beta$ | $< 10^{-3}$ | 0.2 | $< 10^{-3}$ | 0.002 | 0.003 | 0.3 | 0.006 | 0.5 |
| $\gamma$ | 0.02 | 0.3 | 0.03 | 0.02 | 0.03 | 0.8 | 0.05 | 1.0 |

Supplementary Table 11: Continued in next page.

(c) $\mathcal{X}(\mathcal{RA}) > \mathcal{X}(\mathcal{N})$

| | $BC$ | $c$ | $\mathcal{C}$ | $N$ | $E$ | $\beta_0$ | $\beta_1$ | $\beta_2$ |
|---|---|---|---|---|---|---|---|---|
| $B$ | 0.03 | 0.7 | 0.2 | 0.002 | 0.008 | $< 10^{-3}$ | 0.03 | 0.2 |
| $\delta$ | 0.02 | 0.6 | 0.7 | 0.007 | 0.03 | 0.3 | 0.01 | 0.6 |
| $\theta$ | 0.2 | 0.2 | 0.4 | 0.08 | 0.06 | 0.09 | 0.3 | 0.4 |
| $\alpha_1$ | 0.9 | 0.6 | 0.8 | 0.7 | 0.6 | 0.7 | 0.5 | 0.8 |
| $\alpha_2$ | 0.8 | 0.08 | 0.4 | 0.5 | 0.5 | 0.7 | 0.3 | 0.6 |
| $\beta$ | 0.5 | 0.05 | 0.5 | 0.2 | 0.1 | 0.4 | 0.4 | 0.4 |
| $\gamma$ | 0.1 | 0.2 | 0.1 | 0.07 | 0.07 | 0.6 | 0.2 | 0.5 |

(d) $\mathcal{X}(\mathcal{N}) > \mathcal{X}(\mathcal{O})$

| | $BC$ | $c$ | $\mathcal{C}$ | $N$ | $E$ | $\beta_0$ | $\beta_1$ | $\beta_2$ |
|---|---|---|---|---|---|---|---|---|
| $A$ | $< 10^{-3}$ | 0.3 | $< 10^{-3}$ | $< 10^{-3}$ | $< 10^{-3}$ | $< 10^{-3}$ | $< 10^{-3}$ | 0.1 |
| $B$ | $< 10^{-3}$ | 0.2 | 0.005 | 0.003 | 0.004 | 0.2 | $< 10^{-3}$ | 0.5 |
| $C$ | $< 10^{-3}$ | 0.5 | 0.05 | $< 10^{-3}$ | $< 10^{-3}$ | 0.8 | 0.007 | 0.4 |
| $D$ | $< 10^{-3}$ | 0.4 | 0.02 | $< 10^{-3}$ | $< 10^{-3}$ | 0.1 | 0.01 | 0.7 |
| $E$ | $< 10^{-3}$ | 0.7 | $< 10^{-3}$ | $< 10^{-3}$ | $< 10^{-3}$ | 1.0 | $< 10^{-3}$ | 0.7 |
| $F$ | $< 10^{-3}$ | 0.2 | $< 10^{-3}$ | $< 10^{-3}$ | $< 10^{-3}$ | 0.5 | $< 10^{-3}$ | 0.2 |
| $G$ | $< 10^{-3}$ | 0.7 | $< 10^{-3}$ | $< 10^{-3}$ | $< 10^{-3}$ | 0.7 | $< 10^{-3}$ | 0.5 |

Supplementary Table 11: Numerical $p$-values corresponding to the results of panels A-D of Supplementary Figure 4 for the NSF subgroup. These correspond to the group-level comparison between nodes sets, for each considered frequency-band (y-axis) and network metric (x-axis). $\mathcal{X}(S)$ stands for the generalized centrality metric $\mathcal{X}$ measured on the nodes in set $S$.

## S.7. MULTI-FREQUENCY ANALYSIS: INDIVIDUAL PATIENT RESULTS

In this supplementary section we detail the definition of the distinguishability score $\mathcal{D}$ and provide details on the statistical analyses involving this metric. In Supplementary Figure 5 we show the results of the node-based analyses. Each panel corresponds to a frequency band and a comparison between node-sets, as indicated by the panel title. For each panel, we show the result $x_i^{\mathcal{X}}$ of the statistical comparison between the two node-sets, using each of the centrality metrics $\mathcal{X}$ and for each patient $i$ with a color code. The color code indicates whether there is a significant difference in the direction of the hypothesis (blue, $x_i^{\mathcal{X}} = 1$), against it (red, $x_i^{\mathcal{X}} = -1$), or there is no significant difference (grey, $x_i^{\mathcal{X}} = 0$). The patient distinguishability score $D_i$ is simply defined as the sum of the results of this statistical comparison over generalized centrality metrics: $D_i = \sum_{\mathcal{X}} x_i^{\mathcal{X}}$. Given that central nodes have large negative curvature, this term is multiplied by $-1$ in the sum. The resulting patient distinguishability score $D_i$ is thus a number between $-8$ and 8, where $D_i = 8$ $(-8)$ indicates that the two node-sets were highly different in the direction of the hypothesis (against the hypothesis) for all metrics, and $D_i = 0$ indicates no significant or inconsistent differences (across metrics) for the patient.

In Supplementary Figure 6 we show the results of the statistical comparison between the SF and NSF groups based on the distinguishability scores $D_i$, for each of the node-based tests. We observed a tendency towards higher scores for SF patients for the broadband, $\theta$ and $\alpha_1$ bands, and in the opposite direction for $\delta$ and $\gamma$, however none of the differences are significant after FDR correction for multiple comparisons.

Finally, we also performed a receiver-operating-characteristic (ROC) patient-classification analysis based on the distinguishability scores, the results of which were reported in the main text. Here we show in Supplementary Figure 7 the ROC curves corresponding to each of the node-based tests, for the broadband.

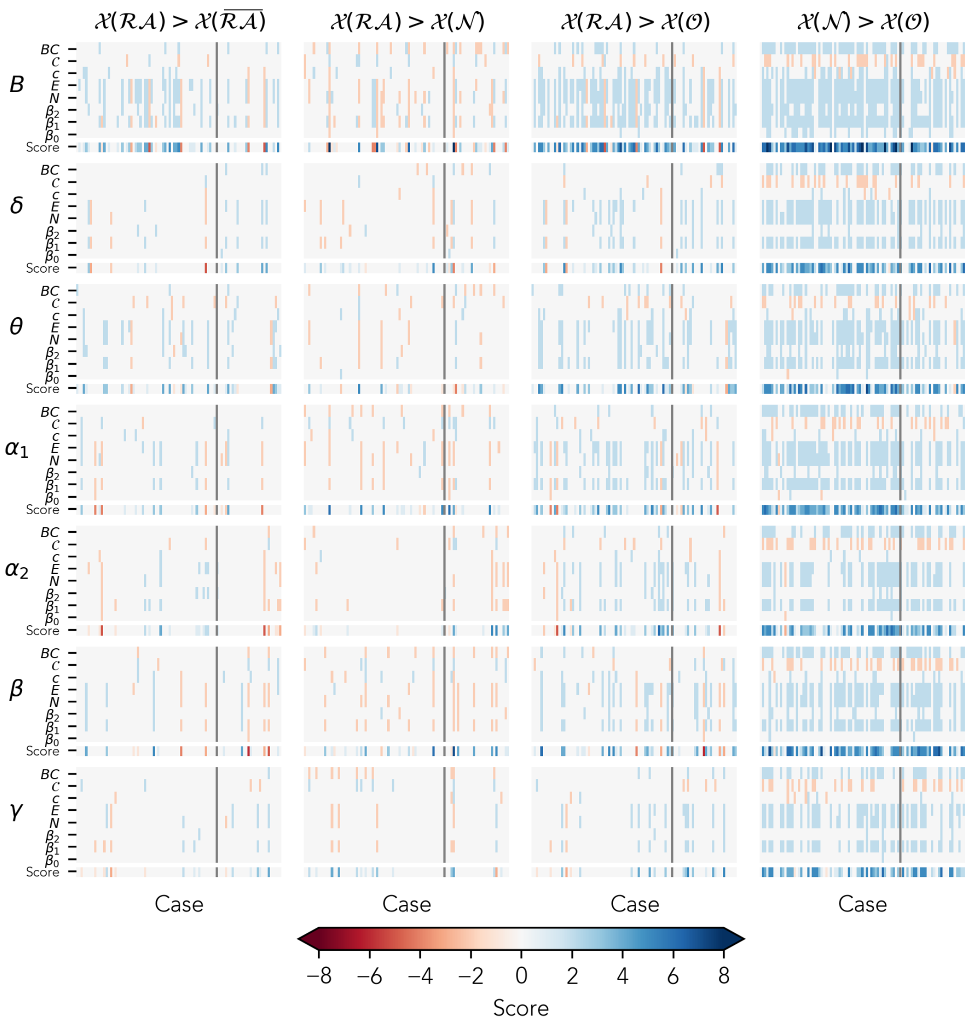


Supplementary Figure 5: Disthinguishability score $D_i$. Each panel corresponds to the comparison between two node-sets as indicated by the panel titles, and a frequency band (from top to bottom: broadband, $\delta$, $\theta$, $\alpha_1$, $\alpha_2$, $\beta$, $\gamma$). We show the result for the statistical comparison $x_i^{\mathcal{X}}$ for each metric $\mathcal{X}$ and each patient $i$ in the top rows of each panel and $D_i$ in the bottom row. All metrics are color-coded as indicated by the colorbar. The vertical grey line on each panel separate SF (left) and NSF (right) cases. Significance (threshold $= 0.05$) was established with a bootstrapping analysis ($n = 10^4$, see Methods). The number of samples in each test was different for each patient since it was given by the size of each node-set. The $p$-values were Bonferroni corrected for multiple comparisons ($n = 56$).

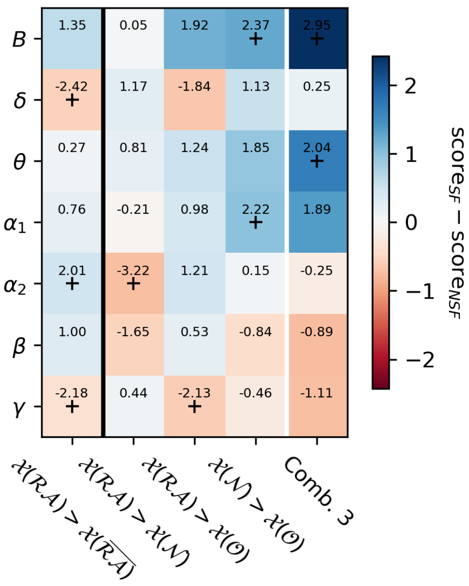


Supplementary Figure 6: Statistical comparison between the SF and NSF patient groups based on the patient distinguishability score $D_i$, for each node-group-comparison (columns) and frequency band (rows), as indicated by the axis labels. The final column combines the results of the three-node-groups tests by adding up the patient scores. The color-code indicates the difference between the average scores of the SF and NSF groups, as given by the color-bar. Significance (threshold = 0.05) was established with a Wilcoxon rank-sum test (see Methods). The t-statistic for each test is shown as a numeric value on each cell. The number of samples in each test was equal to the number of patients on each subgroup: $n_1 = 62$ SF patients and $n_2 = 29$ NSF patients. The $p$-values were FDR corrected for multiple comparisons ($n = 56$). The cross markers indicate differences with $p$-value $< 0.05$ before FDR correction for multiple comparisons. None of the differences were significant after the correction.

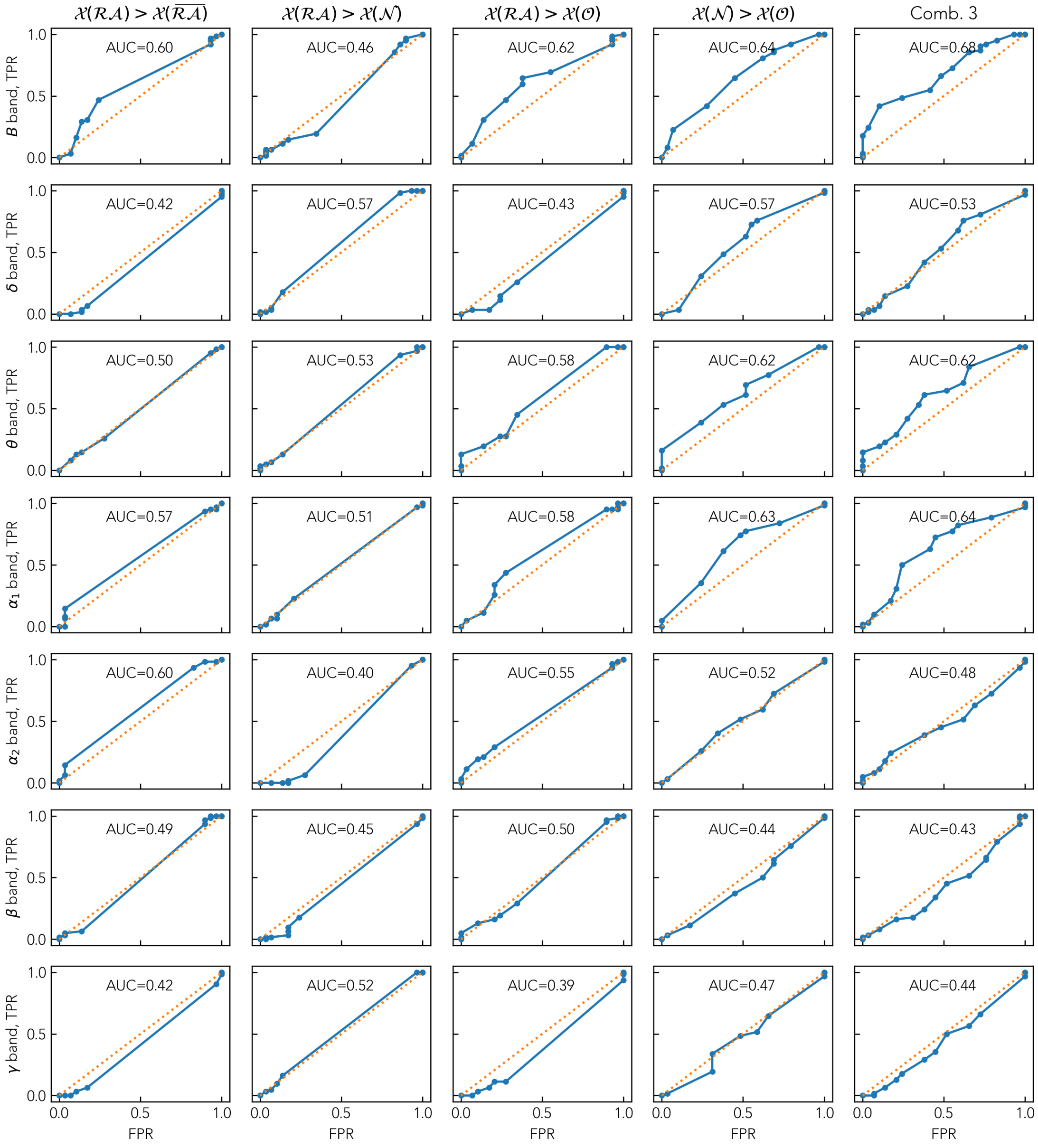


Supplementary Figure 7: ROC analysis of the patient-group classification (SF vs NSF group) based on the patient scores, for each frequency band (rows) and node-group analysis (columns), as shown in Figure 5 of the main text for the broadband. The final column corresponds to the compounded score of the three-node-group analysis. We indicate the area under the curve (AUC) of each curve as the legend. The group sizes were $n_{SF} = 62$, and $n_{NSF} = 29$.

## S.8. SIGNIFICANCE OF THE PATIENT-CLASSIFICATION RESULTS

In Supplementary Table 12 we report the AUC values of the patient classification analyses from which Figure 5c in the main text is derived. For each classification we indicate the AUC of the ROC curve analysis and its 90% confidence intervals resulting from a bootstrapping analysis. $10^4$ bootstrap samples were created by sampling the patient score data with replacement, for each category (SF and NSF) separately. Then $10^4$ bootstrap AUC estimations were produced, from which the confidence interval was derived. To assess whether the AUCs of different tests (i.e. type of comparison and frequency band) differed significantly, we compared the measured AUC of one test against the bootstrap distribution of the other, for all test pairs. Let $B_i$ and $B_j$ denote the sets of bootstrap replicates for tests $i$ and $j$, $i \neq j$, with respective AUCs denoted as $AUC_i$ and $AUC_j$ respectively. We computed the fraction of bootstrap samples $B_i$ smaller than $AUC_j$, and vice-versa the fraction of bootstrap samples $B_j$ smaller than $AUC_i$. The average yielded a two-sided $p$-value for the null hypothesis that there was no significant difference between $AUC_i$ and $AUC_j$. In Supplementary Figure 8 we report the AUC difference between each pair of tests. Significant differences ($p$-value smaller than 0.05 after FDR correction for multiple comparisons) are indicated by a triangular marker.

| | $\mathcal{X}(\mathcal{RA}) > \mathcal{X}(\overline{\mathcal{RA}})$ | $\mathcal{X}(\mathcal{RA}) > \mathcal{X}(\mathcal{N})$ | $\mathcal{X}(\mathcal{RA}) > \mathcal{X}(\mathcal{O})$ | $\mathcal{X}(\mathcal{N}) > \mathcal{X}(\mathcal{O})$ | Comb. 3 |
|---|---|---|---|---|---|
| $B$ | 0.60 (0.50, 0.69) | 0.46 (0.36, 0.57) | 0.62 (0.52, 0.72) | 0.64 (0.54, 0.74) | 0.68 (0.58, 0.77) |
| $\delta$ | 0.42 (0.36, 0.49) | 0.57 (0.48, 0.65) | 0.43 (0.34, 0.51) | 0.57 (0.45, 0.67) | 0.53 (0.42, 0.64) |
| $\theta$ | 0.50 (0.41, 0.59) | 0.53 (0.45, 0.61) | 0.58 (0.48, 0.68) | 0.62 (0.52, 0.71) | 0.62 (0.52, 0.72) |
| $\alpha_1$ | 0.57 (0.50, 0.63) | 0.51 (0.42, 0.59) | 0.58 (0.48, 0.68) | 0.63 (0.52, 0.72) | 0.64 (0.53, 0.74) |
| $\alpha_2$ | 0.60 (0.52, 0.67) | 0.40 (0.32, 0.49) | 0.55 (0.47, 0.63) | 0.52 (0.40, 0.62) | 0.48 (0.37, 0.58) |
| $\beta$ | 0.49 (0.41, 0.57) | 0.45 (0.37, 0.54) | 0.50 (0.40, 0.59) | 0.44 (0.34, 0.55) | 0.43 (0.33, 0.53) |
| $\gamma$ | 0.42 (0.35, 0.49) | 0.52 (0.45, 0.60) | 0.39 (0.32, 0.47) | 0.47 (0.37, 0.57) | 0.44 (0.34, 0.55) |

Supplementary Table 12: Numerical AUC values of the patient classification analysis (SF vs NSF) for each frequency band (rows) and type of node comparison (columns), corresponding to the results shown in Figure 5 of the main text. We report the AUC of the classification together with 90% confidence intervals (within brackets) derived from the bootstrapping analysis ($n = 10^4$).

## S.9. EFFECT OF THE RESECTION TYPE

We analyzed whether there were differences between patients with temporal versus extra-temporal resections. In order to do so, we repeated the analyses of Figure 5 of the main text independently for the temporal ($n = 61$, see Supplementary Table 1) and extra-temporal ($n = 30$) subgroups. The results are summarized in Supplementary Figure 9. Firstly, we have performed the patient-classification analysis (to classify the patients into the SF and NSF groups) independently for the temporal and extra-temporal subgroups. The resulting AUC values for each frequency band and type of comparison are shown respectively in panels A and B of the figure. The temporal subgroup displays the same behavior as the full group (as expected since it comprises just over two thirds of all cases). Some variations are observed for the extra-temporal subgroup, particularly with larger AUC values for the Combined test in the $\theta$-band. To identify whether there were significant differences between the temporal and extra-temporal subgroups, in panel C we show the box-plot comparison of the AUC values. On average, the AUC was slightly larger for the extratemporal (average$= 0.51$, 90% confidence interval$= (0.39, 0.65)$) than for the temporal subgroup (average$= 0.47$, 90% confidence interval$= (0.37, 0.56)$). The difference was significant according to the Wilcoxon

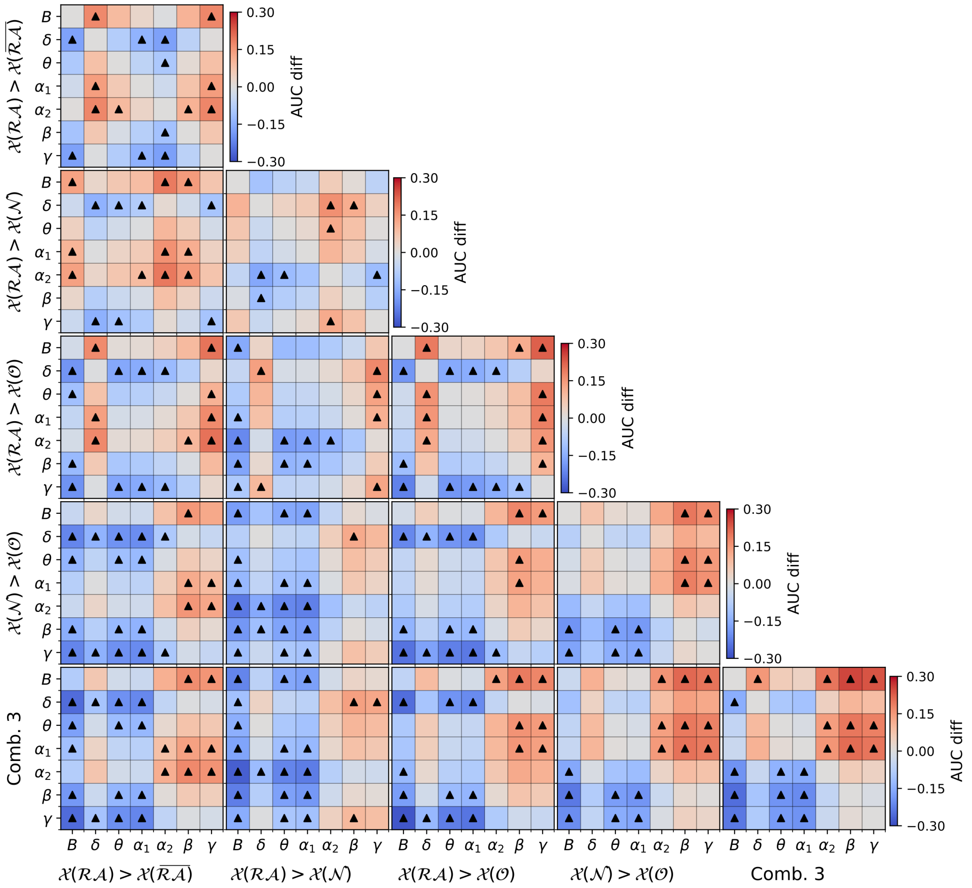


Supplementary Figure 8: Difference in AUC values of the patient classification (SF vs NSF) between different tests (frequency band and comparison type). Each panel corresponds to a pair of node-comparison type (the first test is given by the panel row and the second by the panel column). In each panel, we show the difference between all frequency-band pairs, defined as AUC diff $= AUC_1 - AUC_2$ (the first frequency band is given by the rows and the second by the columns). Significant differences (according to Bootstrap analyses, see Methods, $n = 10^4$) are shown by triangular markers (corrected $p$-value smaller than 0.05 after FDR correction). In all panels, $n_{SF} = 62$, $n_{NSF} = 29$.

signed-rank test for paired variables ($p$-value$= 0.03$, Wilcoxon statistic $= 185.0$, sample size $n = 35$, effect size $r = -0.36$), albeit the difference was small ($AUC$(temporal) $-$ $AUC$(extra-temporal) $= -0.04$).

We also compared the patient-classification results (between the temporal and extra-temporal subgroups) at the individual test level, with the same bootstrapping analysis as described in the previous section. We found that the extra-temporal subgroup presented

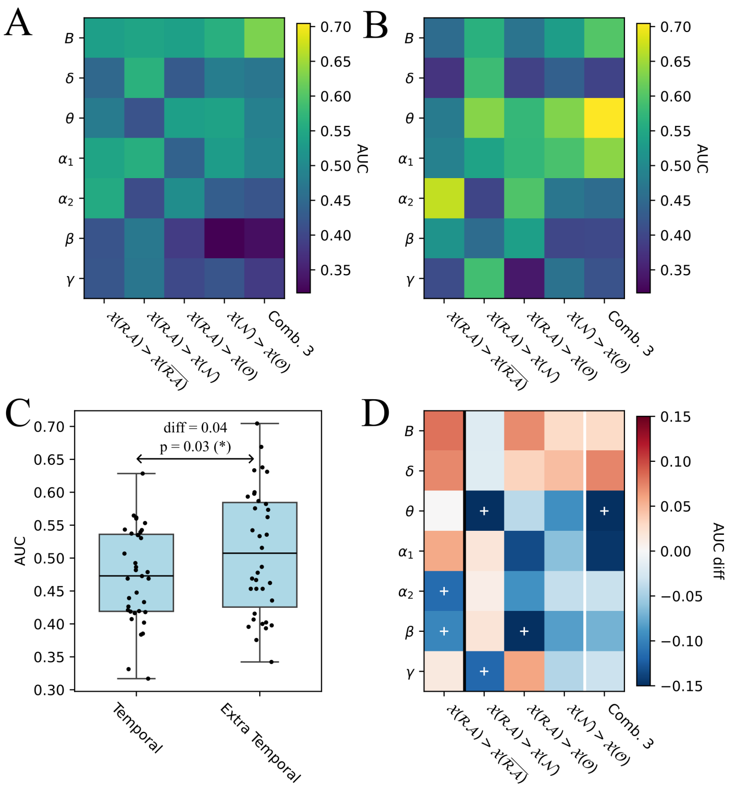


Supplementary Figure 9: Comparison between the temporal and extra-temporal subgroups. Panels A and B show the AUC of the patient outcome classification (SF vs NSF) respectively for the temporal and extra-temporal subgroups. Panel C shows the box-plot of these values for each group, and the results of the Wilcoxon signed-rank comparison, which yields that the temporal subgroup presents a significantly lower AUC on average than the extra-temporal subgroup ($W = 185.0$, $n = 35$, $r = -0.36$). Data-points represent individual patients. Panel D shows the results of the individual-test comparison. The color-code indicates the AUC difference between the temporal and extra-temporal subgroups (AUC $diff = AUC(\text{temporal}) - AUC(\text{extra-temporal})$), as indicated by the colorbar. Significant differences (bootstrap analysis, $n = 10^4$) are shown by the white cross markers. None survived FDR correction for multiple comparisons ($n = 35$).

significantly larger $AUC$ results for some individual tests (white cross markers in panel D of the figure), but these did not survive FDR correction for multiple comparisons.

## S.10. ALTERNATIVE DISTINGUISHABILITY SCORE

In order to compare our findings with a recent study by Ramaraju and colleagues [4], we repeated the patient-classification analysis using their original definition of the distinguishability score, $D'_i$. We also consider the same centrality metric used by [4], the weighted degree or strength of a node (the sum of its link weights after thresholding the PLI matrix with the disparity filter). The distinguishability score for each patient $D'_i$ was defined by [4] as the AUC of the ROC-classification analysis of the $\mathcal{RA}$ and $\overline{\mathcal{RA}}$ node sets. A $D'$ value close to 0.5 indicates that the two node sets cannot be classified according to the corresponding metric, whereas values close to 0 or 1 indicate that the node sets are easily classifiable. In particular, $AUC > 0.5$ indicates that the $\mathcal{RA}$ set is more central than the $\overline{\mathcal{RA}}$ set, and vice versa for $AUC < 0.5$. The results of this analysis are shown in Supplementary Table 13 (first column).

We did not find a good patient classification for any frequency band. The best classification results were obtained for the $\gamma$ band with $AUC = 0.64$, followed by the $\alpha_1$ ($AUC = 0.39$) and $\alpha_2$ ($AUC = 0.60$) bands. Interestingly, the direction of the classification changed across frequency bands: for $\alpha_2$ and $\gamma$ SF patients presented higher distinguishability scores $D'_i$ than NSF patients, whereas for $\alpha_1$ the opposite was true.

In order to exploit the three-node-set partition framework defined in the main text, we extended this analysis to account for three more two-class node-based classifications, namely i) $\mathcal{RA}$ and $\mathcal{N}$ nodes; ii) $\mathcal{RA}$ and $\mathcal{O}$ nodes; and $\mathcal{N}$ and $\mathcal{O}$ nodes (Supplementary Table 13). Swarm plots depicting distinguishability scores are presented in Supplementary Figure 10 for the case of broad band for a visual representation of the classification. We found that the results for the latter two cases were very similar to the original $\mathcal{RA}$ and $\overline{\mathcal{RA}}$ distinguishability. As expected from the results in Figure 3 in the main text, the $\mathcal{RA}$ and $\mathcal{N}$ cannot be easily classified, resulting in low node-distinguishability scores and in a poor patient classification.

Finally, we repeated this analysis on our proposed framework of 8 generalized centrality metrics, the results are shown in Supplementary Figure 11. The results were similar to those using the weighted degree, with only fair patient classification results. The best findings were obtained when considering the two-node-set partition (i.e. $\mathcal{RA}$ versus $\overline{\mathcal{RA}}$) in the broadband ($AUC = 0.68$ for the metric $E$), and overall showed large variability also in the direction of

| | $\mathcal{X}(\mathcal{RA}) > \mathcal{X}(\overline{\mathcal{RA}})$ | $\mathcal{X}(\mathcal{RA}) > \mathcal{X}(\mathcal{N})$ | $\mathcal{X}(\mathcal{RA}) > \mathcal{X}(\mathcal{O})$ | $\mathcal{X}(\mathcal{N}) > \mathcal{X}(\mathcal{O})$ |
|---|---|---|---|---|
| $B$ | 0.56 | 0.51 | 0.57 | 0.49 |
| $\delta$ | 0.57 | 0.56 | 0.59 | 0.57 |
| $\theta$ | 0.42 | 0.46 | 0.43 | 0.44 |
| $\alpha_1$ | **0.39** | 0.41 | **0.40** | 0.51 |
| $\alpha_2$ | **0.60** | 0.55 | **0.61** | **0.60** |
| $\beta$ | 0.48 | 0.47 | 0.45 | 0.50 |
| $\gamma$ | **0.64** | 0.58 | **0.65** | 0.57 |

Supplementary Table 13: Results of the patient classification following the methodology in [4]. We report the area under the curve (AUC) of the patient classification (SF versus NSF) based on the distinguishability $D'$ between $\mathcal{RA}$ and $\overline{\mathcal{RA}}$ nodes (first column), when using the weighted degree as nodal centrality metric. $\mathcal{X}(S)$ stands for the generalized centrality metric $\mathcal{X}$ measured on the nodes in set $S$. The latter three columns extend this analysis to the three-node-set framework by considering the distinguishability between the i) $\mathcal{RA}$ and $\mathcal{N}$ nodes; ii) $\mathcal{RA}$ and $\mathcal{O}$ nodes; and $\mathcal{N}$ and $\mathcal{O}$ node sets, respectively. Each row corresponds to a different frequency band. We highlight in bold the results for with $|AUC - 0.5| > 0.1$.

the $AUC$ (that is, whether SF or NSF patients presented larger distinguishability scores). Thus this extended analysis was not able to improve upon our initial results.

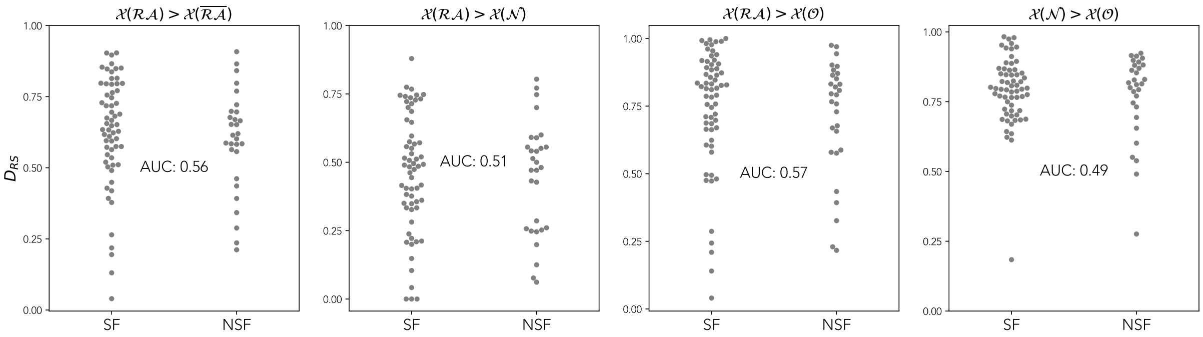


Supplementary Figure 10: Swarm plot depicting distinguishability values ($D'_{RS}$) for SF and NSF surgical outcomes for different node-based comparisons, based on the node strength. $\mathcal{X}(S)$ stands for the generalized centrality metric $\mathcal{X}$ (node strength here) measured on the nodes in set $S$. Values close to 0 (1) indicate that high strength nodes are resected (spared). Each scatter point represents an individual patient. We indicate the AUC of the ROC patient-classification analysis ($n_{SF} = 62$, $n_{NSF} = 29$). The AUC results for every band are reported in Supplementary Table 13.

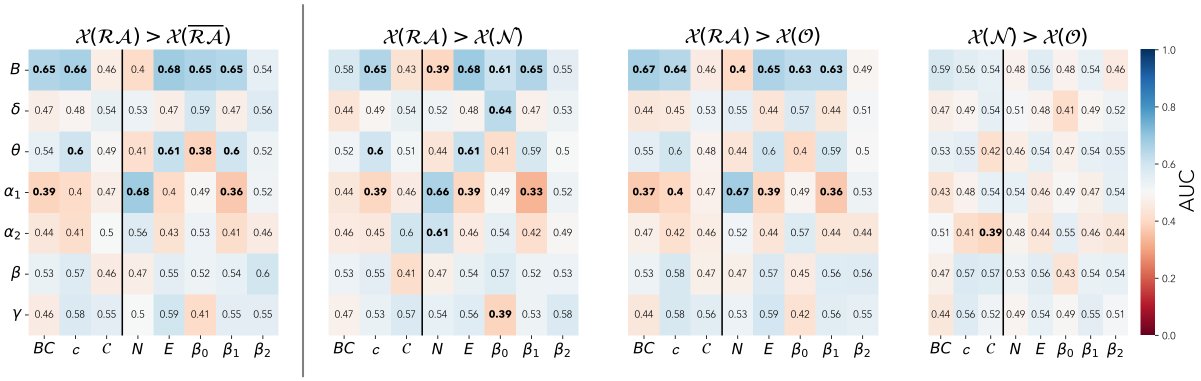


Supplementary Figure 11: Patient classification results (SF vs NSF) using the methodology by [4] combined with our proposed generalized centrality metrics. Each panel corresponds to a node-based comparison as indicated by the panel titles, with the vertical line separating the two-node-set case from the three-node-set cases. $\mathcal{X}(S)$ stands for the generalized centrality metric $\mathcal{X}$ measured on the nodes in set $S$. Rows correspond to frequency bands and columns to generalized centrality metrics. We show the resulting AUC of the ROC classification analysis ($n_{SF} = 62$, $n_{NSF} = 29$) both with the color-code and by numerical values. Bold numbers correspond to $|AUC - 0.5| > 0.1$.

## SUPPLEMENTARY REFERENCES


[1] M. Newman, *Networks* (Oxford university press, 2018).

[2] F. A. Santos, E. P. Raposo, M. D. Coutinho-Filho, M. Copelli, C. J. Stam, and L. Douw, Topological phase transitions in functional brain networks, Physical Review E **100**, 032414 (2019).

[3] A. P. Kartun-Giles and G. Bianconi, Beyond the clustering coefficient: A topological analysis of node neighbourhoods in complex networks, Chaos, Solitons & Fractals: X **1**, 100004 (2019).

[4] S. Ramaraju, Y. Wang, N. Sinha, A. W. McEvoy, A. Miserocchi, J. De Tisi, J. S. Duncan, F. Rugg-Gunn, and P. N. Taylor, Removal of interictal meg-derived network hubs is associated with postoperative seizure freedom, Frontiers in neurology **11**, 563847 (2020).
